# Review of Polymer Composites with Diverse Nanofillers for Electromagnetic Interference Shielding

**DOI:** 10.3390/nano10030541

**Published:** 2020-03-17

**Authors:** Dimuthu Wanasinghe, Farhad Aslani, Guowei Ma, Daryoush Habibi

**Affiliations:** 1 School of Engineering, University of Western Australia, Crawley WA 6009, Australia; dimuthu.wanasinghe@research.uwa.edu.au (D.W.); guowei.ma@uwa.edu.au (G.M.); 2 Materials and Structures Innovation Group, School of Engineering, Edith Cowan University, Joondalup WA 6027, Australia; d.habibi@ecu.edu.au

**Keywords:** EMI, shielding, polymer, composites, nanomaterials

## Abstract

Polymer matrix composites have generated a great deal of attention in recent decades in various fields due to numerous advantages polymer offer. The advancement of technology has led to stringent requirements in shielding materials as more and more electronic devices are known to cause electromagnetic interference (EMI) in other devices. The drive to fabricate alternative materials is generated by the shortcomings of the existing metallic panels. While polymers are more economical, easy to fabricate, and corrosion resistant, they are known to be inherent electrical insulators. Since high electrical conductivity is a sought after property of EMI shielding materials, polymers with fillers to increase their electrical conductivity are commonly investigated for EMI shielding. Recently, composites with nanofillers also have attracted attention due to the superior properties they provide compared to their micro counterparts. In this review polymer composites with various types of fillers have been analysed to assess the EMI shielding properties generated by each. Apart from the properties, the manufacturing processes and morphological properties of composites have been analysed in this review to find the best polymer matrix composites for EMI shielding.

## 1. Introduction

Recent decades have seen rapid growth in the use of consumer electronics, primarily due to consumer demand and the advancement of technology. While this demand-driven growth has made the lives of the consumers more comfortable, it has also generated inadvertent harmful effects. Emission of electromagnetic waves (EMWs), during the operation of these devices, is one such harmful effect. While EMWs can be found in Nature in the form of light, many electronic devices emit EMWs, which are of frequencies that are not found naturally [1,2,3,4]. Some research into prolonged exposure of high-frequency EMWs have shown that they can affect human health, leading to complicated illnesses [5,6,7,8,9,10,11,12,13]. However, the most common problem with the EMWs generated by such electronic devices is their interaction with other electronic devices. These interactions are deemed unwanted since EMWs can induce an electrical current in a conductor which could interfere with the functionality of the second device [14,15,16,17,18,19]. This phenomenon is well known, so the protection of electronic devices is of paramount importance in order to maintain their proper functionality. In some instances, artificially generated EMI can be used as a weapon that could cripple the electronic systems, such as the threat posed by a high altitude electromagnetic pulse (HEMP). For this reason, the US Department of Defence has defined an EMI shielding limit for critical buildings to prevent damage to any electronic equipment stored within them [20,21,22,23,24,25,26]. Additionally, buildings such as hospitals have special requirements for EMI shielding since they house equipment which emit EMWs of varying frequencies and also equipment which is extremely sensitive to EMI [27,28,29,30,31,32,33,34].

The disruption caused by the EMWs from one device in a second device is known as the electromagnetic interference (EMI) [14,35,36]. Many of household electric appliances have sufficient protection to prevent any harmful effects that can be created within them. Traditionally, these protection enclosures have been fabricated with metals, which is the most commonly used material in fabricating EMI shields [29,37]. The primary reason for using metals for EMI shielding enclosures is their high electrical conductivity. High conducting materials are good EMI shields because they form a Faraday cage when encountering EMWs, causing the charges to be induced on the enclosure without disrupting the functionality of the components inside. Metals are excellent in terms of creating such EMI shields but have drawbacks in their physical and corrosion-resistant properties [38,39,40]. The best way to overcome the drawback is to fabricate the shields from alternative materials [41]. Most of the alternative materials do not have as much conductivity as metals and as a result, cannot form a Faraday cage to prevent EMI.

Much of the research on alternative materials that can be used for EMI shielding has also seen an increase due to the growth in the demand. The number of publications on alternative materials for EMI shielding has shown an exponential rise [42]. However, many of these publications show that the new materials being researched do not meet the shielding efficiency (SE) needed in electronic equipment. Apart from corrosion properties, the other main reason for the search for alternative materials for EMI shielding is the miniaturisation of equipment, which leads to more stringent shielding requirements. For these reasons, polymers have chosen as the ideal material. However, polymers are not electrical conductors, which poses a problem in developing them for EMI shields. To impart electrical conductivity within polymers, different additives with high electrical conductivity can be added and at the same time these additives enhance the electrical conductivity, they can also improve the mechanical properties.

Many of the fillers that are being used in these polymer composite include carbon nanotubes which are known to have exceptionally high electrical conductivity. Other fillers include particles which have been coated with silver, metal fibres, and carbon particles [15,43,44,45,46]. Many of the studies conducted on the fabrication of EMI shielding materials have shown that for high SE, a good conducting network needs to be present within the material. Even with a good conducting network, some of the EMWs can penetrate the surface of the material and enter the material. To reduce the EMWs that penetrate the material, additives should be in place to absorb or attenuate EMWs by subjecting them to multiple reflections [47]. Many of the research on EMI shielding polymeric materials have focused on achieving one of these goals in order to prevent EMWs from passing through to the other side of the material. Being an insulating material, polymer cannot reflect EMWs on their own and requires high conductive filler to reflect them.

## 2. Theory of Electromagnetic Shielding

**Figure 1:**
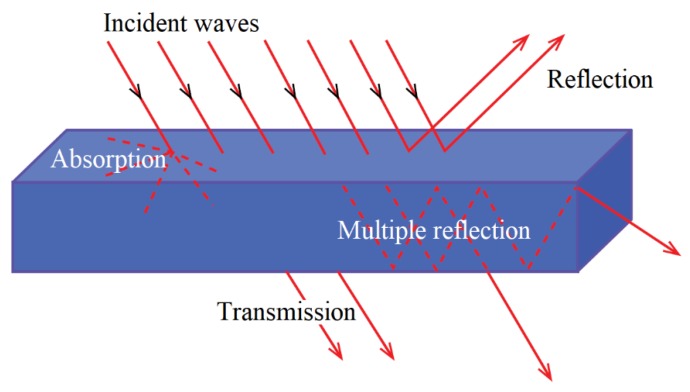
Schematic representation showing the mechanism of electromagnetic shielding. Reproduced with permission from Ajay et al. [48], Copyright 2018, John Wiley and Sons.

EMWs undergo several interactions when they intercept different materials. Depending upon the conductivity and the relative permeability, EMWs can be reflected, absorbed, or attenuated within the material, as illustrated in Figure 1 [48]. If the EMWs do not undergo such or only partially undergo such interactions, the balance amount passes through the material. The amount of EMWs that passes through can cause interference within electronic components on the other side. To minimise these interactions, the maximum number of EMWs need to be prevented from going through the material.

When an EMW encounters a material, the interaction between them leads to a generation of current in the material and ohmic losses resulting in the EMW to lose its intensity. The distance from the top of the material to reduce the strength of the EMW to *1/e* of its original strength is known as the skin depth (*δ*), which can be calculated as follows [49]:

(1) $$\delta =\frac{1}{\sqrt{\pi f\mu \sigma }}$$

In Equation (1), *f* is the frequency of the EMW, *μ* is the relative permeability, and *σ* is the electrical conductivity. Several theories are used to calculate the SE of material, including Plane-Wave Theory, Metal foil, Near Field Shielding, Low-Frequency Magnetic Field Source, and Scattering Parameter. Out of these theories, the Plane-Wave Theory is the most commonly used for the calculation of SE based on the properties and the thickness of the material. There are three mechanisms that contribute to the total shielding mechanism, which are known as reflection, absorption, and multiple reflections.

The absorption in the Plane-Wave Theory is calculated as follows, where *t* is the thickness of the specimen:

(2) $$SE_{A}=20\log e^{-\left(t/\delta \right)}$$

For the calculation of the reflection loss, first, the intrinsic impedance of the material (*η*_s_) need to calculated as shown in Equation (3). Afterwards, the SE, due to reflection, can be calculated, as shown in Equation 4, where *η_0_* is the impedance of free space:

(3) $$\eta_{s}=\sqrt{\frac{2\pi f\mu }{\sigma }}$$

(4) $$SE_{R}=20\log\frac{\eta_{0}}{4\eta_{s}}$$

For single-layer materials, the total shielding effect would be due to reflection and absorption:

(5) $$SE=20\log\frac{\eta_{0}}{4\eta_{s}}+20\log e^{\left(t/\delta \right)}$$

However, for materials with multiple thin layers, the totals shielding effect is the contribution of the reflection, absorption, and multiple reflections, which is shown in Equation (6) [50,51]:

(6) $$SE_{T}=20\log\frac{\eta_{0}}{4\eta_{s}}+20\log e^{\left(t/\delta \right)}+20\log\left|1-e^{-2t/\delta }\right|$$

For thin metallic sheets, the calculation of the total SE needs to be carried out using the metal foil method. While there are several methods for the calculation of the theoretical SE, some of these methods go concurrently with the actual methods used to measure the SE. There are several methods developed for the measurement of the EMI SE based on the type of material and the frequency range. The four most commonly used SE measurement methods are:

(1) Open field or free space test
(2) Shielded box test
(3) Coaxial transmission line test
(4) Shielded room test

Out of the four methods listed, the coaxial transmission line method is one of the most commonly used methods since the results of this technique are repeatable and can be used for a wide range of frequencies. The scattering parameter method is used to calculate the SE theoretically for the coaxial transmission line technique [52,53]. The open-field method is generally used to measure the SE of an entire assembly as an antenna placed in the open space measures the amount of radiation emitted from the electronic device that is kept within a certain distance from the antenna [20,43,54]. The shielded box and room tests are somewhat similar techniques with minor differences. In both methods, the specimen is irradiated from one antenna and the EMWs coming out from through the specimen with the second antenna. The type of method that can be used for the measurement would depend on many factors, such as the frequency of the testing, type of material, and specimen geometry. For the generation and the measurement of the EMWs that passes through and reflected from the specimen, a Vector Network Analyser is generally used [55,56]. 

While these techniques are effective methods for the measurement of SE of the fabricated material, other testing techniques are necessary for the measurement of properties which are present in these materials. One of the main reasons to use polymer composites to replace metallic shields that are currently used is the corrosion-resistant of the polymeric composites. For this reason, it is necessary to measure the degradation of the polymers. Additionally, morphological characterisation techniques such as scanning electron microscopy (SEM) and transmission electronic microscopy (TEM) is necessary to observe the distribution of the fillers that are used to enhance the shielding properties. For additional characterisations, techniques such as Fourier Transformation Infrared (FTIR) spectroscopy, tensile tests, and X-ray diffraction (XRD) can be used to assess the chemical composition and the mechanical properties of the composites [57]. 

## 3. Polymer-Based EMI Shielding Materials

With the evolution of the electronics industry, there has been a rise in pollution created by the EMWs in the atmosphere. Hence, there has been an increasing need to make new EMI shielding material since conventionally used metals are prone to corrosion, expensive, and difficult to be manufactured into complex shapes. Polymers ideally suit these requirements since polymers are low in cost, corrosion-resistant, low in density, and easy to manufacture [47]. However, polymers being natural insulators are not very good in creating an effective shield against EMI on their own [58]. Hence, they need to be mixed with other materials to be made into a composite to be suitable shielding material.

Many different fabrication techniques are used for the fabrication of polymer matrix composites for EMI shielding. Many of these techniques are same as the ones being used for the traditional polymer composites, which involves coating the fibres or particles with the polymer resin and curing to achieve the final strength. In addition to being economical, this technique is also known to provide adequate strength to the composite to withstand mechanical forces it would be subjected to [59]. However, the stringent property requirements of EMI shielding composites requires careful control of the manufacturing atmosphere and purity of the chemical being used [60,61]. Additionally, the advancement of technologies have introduced novel manufacturing techniques such as 3D printing. As 3D printing can help to simplify the manufacturing process, it is currently being employed in many polymer studies [62]. Many different fabrication techniques have been employed for the polymer matrix composites reviewed in this work, which are discussed briefly. 

A study was conducted to evaluate the SE of 3D printed pyramidal-shaped layers of polyamide in the frequency range of 0.1 to 0.3 THz. The results obtained from this test have shown that the attenuation of the EMWs from the polymer is very low and depends on the angle of the incident waves falling on the polymer. Some 60–98% of the incident EMWs are known to be transmitted through these materials depending upon the angle of the incident wave [63]. The low SE is mainly due to the low conductivity of the polymer. This has been the main reason why different additives have been added to polymer matrices to form a polymer-based composite that is being developed for EMI shielding application.

### 3.1. Conducting Polymers 

Even though most polymers are considered to be inherent insulators, there are some polymers which conduct electricity. These have attracted attention in EMI shielding applications since the composite manufactured using conductive polymers would have higher SE compared to conventional polymer matrix composites. Polypyrrole (PPy) is one of the most studied polymers for its high conductivity [64]. PPy can be manufactured easily and is used in fuel cells, corrosion protection applications, computer displays, and as a smart material in biomedical applications [65]. One significant disadvantage of PPy is that after the polymer has been formed, it becomes hard and brittle, acting in a non-thermoplastic manner [66]. Polyaniline (PANI) is another conductive polymer which is being studied for use in many electronic applications [67]. PANI is manufactured using a variety of monomers and can achieve high conductivity since they can be doped with other materials [68]. The conductivity of PANI results from the ability of the charge carriers that were introduced during doping, to move along the polymer chain [69,70]. Even though the conductivity of PANI is not as high as that of metals, it is considerably high compared to the conductivity of other polymers. Additionally, PANI has many advantages, including ease of fabrication, low cost, and the ability to be switched between electrically conducting and insulating status [66,71].

Because of its considerably good conductivity, PANI has been used in some polymer composites for EMI shielding. One of the early usages of PANI has been in the form of a thin coating on polyester cloth, glass fabrics, and high silica cloth that has enabled the fabrication of a thin, lightweight, and flexible EMI shielding composites. Out of the three types of clothes used, glass fabric-coated PANI has shown the highest conductivity. SE tests conducted within the 100 to 1000 MHz frequency range have shown that these composites can provide a SE of 30 to 40 dB [72]. In a similar study, PPy has been coated on nylon-6 and tested for the SE. The frequency range that has been used for the EMI shielding tests in this research was 0.4 to 10 GHz. The composite has shown a SE of about 35 dB in this frequency range. The authors have also tested the SE while varying the thickness of the specimen, which has shown that the overall SE increases with thickness due to the increased absorption of EMWs by the composite [73]. 

A composite consisting of a butyl rubber matrix and low-density polyethene fillers has been synthesised and tested for EMW shielding and conductive properties. The composite has been tested within 1 to 16 GHz frequency range. A composite with 10 wt% of fillers has shown the best SE, which is about 50 to 60 dB. The SE of the composite has shown a decrease with the increase of the frequency. The current and voltage characteristics of the composite have shown current switching characteristics [74].

**Figure 2:**
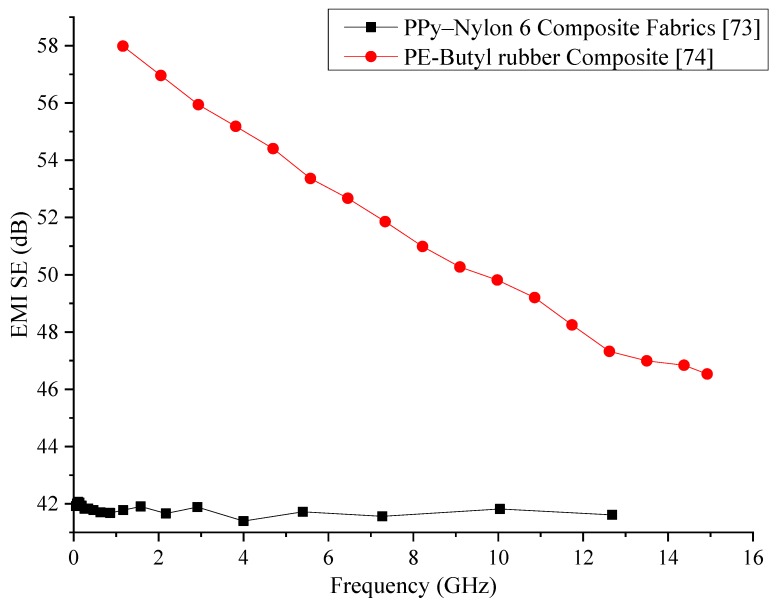
EMI SE comparison of reviewed conductive polymer composites.

Data of the electrically conductive polymers reviewed in this section is provided in Table 1. Comparison of the conductive polymers reviewed in this section has been graphically represented in Figure 2 by using the data provided in the respective publications. From a first glance at the comparison, it can be seen that the polyethene/butyl rubber composite has much better SE than the PPy/nylon composite. However, the SE produced by the PE/rubber composite shows a reduction with increasing frequency while the PPy/nylon composite manages to maintain its SE at a relatively constant value. Further development of the PE/rubber composite with the inclusion of other wave absorbers could be useful in enhancing the SE of this composite that would produce a better shielding behaviour through the entire frequency of testing. Regardless, since high SE is the requirement of the industry, it is recommendable that the PE/rubber composite be adopted for industrial use. Additional mechanical property analysis would have provided additional details about the two composites that would have been able to assess the suitability of the two composites in replacing existing metal sheets for EMI shielding.

### 3.2. Carbon Black-Based Polymer Composites

**Figure 3:**
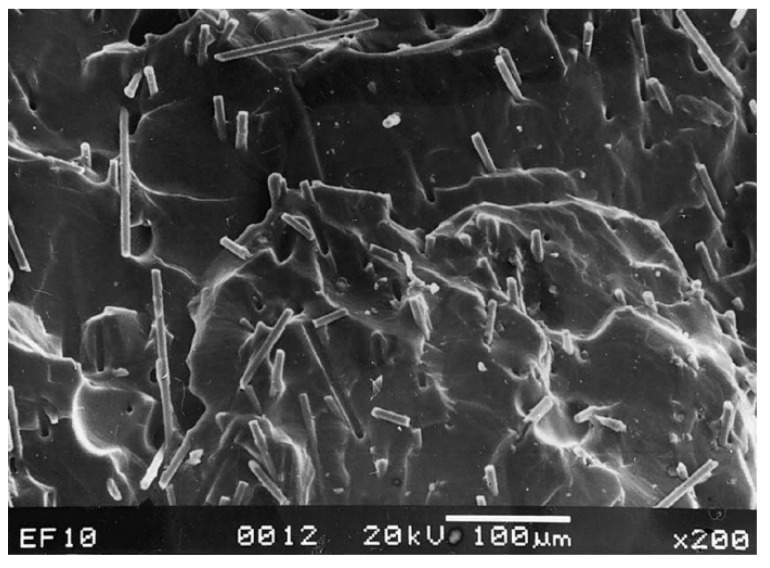
SEM images showing (**a**) the distribution of SCF within the EVA matrix. Reproduced with permission from Das et al. [75], Copyright 2000, Elsevier Ltd.

Carbon, being an excellent conductor, has been the ideal choice to be used for polymer-based composites to enhance the electrical conductivity and EMI SE. An experiment to measure the SE of conductive carbon black and short carbon fibres (SCF) with natural rubber and ethylene-vinyl acetate (EVA) as the matrix material have been carried out in the frequency ranges of 100 to 2000 MHz and 8 to 12 GHz. The SEM analysis conducted on the specimen, shown in Figure 3, has shown the proper distribution of the SCF within the two matrices. The results from the shielding test have shown that out of the two filler materials used, the SCF has better shielding and out of the two matrices, EVA has better shielding properties which are above 20 dB for the 8 to 12 GHz frequency range [75]. Subsequent research has looked into how EVA matrix-based composites consisting of conductive carbon black, SCF, and MWCNT perform as EMI shields over the frequency range of 7.8 to 12.4 GHz. The results have shown that carbon black-based composite has lower SE than the other two systems while SCF based system has the highest SE while MWCNT tends to break into smaller pieces during the dry mixing process leading to a lowered shielding properties than anticipated [76].

Nylon 6,6- and polycarbonate matrices-based composites consisting of electrically conductive carbon black, synthetic graphite particles, and milled pitch-based carbon fibre have been fabricated by extrusion and injection moulding and tested for their EMI SE. In addition to creating composites with these carbon additives individually, the study has investigated the SE of composites containing a mixture of these additives as well. The SE has been measured for the frequency range of 30 MHz to 1.5 GHz. Through the testing, they have found that the length and aspect ratio of the carbon fibre gets reduced due to the extrusion and the injection moulding processes. The SE has found to be highest in the composite containing carbon black, followed by graphite particles and carbon fibres. For the tested frequency range, a SE of 40 to 42 dB has been the highest value obtained by any of the composites. In the composites containing the mixes, the composite with all the three additives has shown outstanding SE due to the creation of good 3D conducting network [77].

Nanocarbon black (CB) particles with an average particle size of 45 nm have been mixed into commercially available resole-type phenolic resin to be made into an EMI shielding composite material. Additional testing conducted on the composites has shown that the addition of CB nanoparticles has enhanced the thermal stability of the composites as well. The SE of the composite over the frequency range of 8 to 12 GHz has shown a range of 30 to 40 dB, which shows that this type of composites could have potential applications in the industry [78]. Even though this composite provides good SE for the measured frequency range, the cost of producing nanoparticles make the cost of the entire composite to increase. 

To fabricate a CB/polymer composite for EMI shielding which is cost-effective, general-purpose furnace (GPF) carbon black has been mixed in with natural rubber/butyl rubber (70/30). The synthesised composite has shown an EMI SE of 7 to 30 dB within the frequency range of 0.5 to 5.0 GHz. The test results have shown that the SE of the composite increases with the increase of the CB loading in the matrix. In addition to the shielding properties, thermal stability, electrical conductivity, and tensile strength of the composite have also increased due to the addition of CB particles [79]. To test the EMI SE of different carbon additives on polycarbonate (PC) matrix, composites containing carbon black, carbon nanotubes, and graphene nanoplatelets have been fabricated and tested within the frequency range of 8.5 to 12 GHz. The SE of these composites has shown maximum values of 12, 13 and 11 dB, respectively. Apart from this finding, they have also been able to conclude that having high electrical conductivity does not necessarily generate good EMI SE in these composites, but parameters such as carbon additive concentration, sample thickness, the incident angle of EMWs, and frequency of EMWs are more critical factors to be considered for high SE [80]. 

Microcrystalline graphite (MCG) is a form of natural graphite which has a high degree of graphitisation and consists of microcrystals with natural orientations [81]. A composite with MCG filler in a low-density polyethene (LDPE) matrix has been tested for the EMI SE due to good EMW absorbing properties of MCG. A sample which is 2.0 to 2.1 mm thick has been subjected to EMWs with a frequency range of 2 to 18 GHz. The results have shown an EMI reflective peak of 20.46 dB at 3.02 GHz. Overall results from this experiment have shown that MCG has the potential to be used as an effective filler in EMI shielding composites with further improvements [82]. To create a composite with a lower content of filler material the use of Ketjen carbon black (K-CB), which is a superior form of conductive carbon black, has been added to chlorinated polyethene (CPE) and tested for its EMI SE in the frequency range of 8.2 to 12.4 GHz. The microstructure observed through SEM has shown good dispersion of the particles within the matrix. The electrical conductivity of the composite has shown a sharp rise when the filler content is increased initially from 0 to 15%, whereas afterwards, it has not shown a significant increase. Composite with 30% K-CB and 1 mm thickness has shown a SE of 38.4 dB, which is a considerably high value for a composite with CB as the filler. Additionally, this research has proven that it is possible to fabricate a composite with a high SE at a considerably low cost [83].

**Figure 4:**
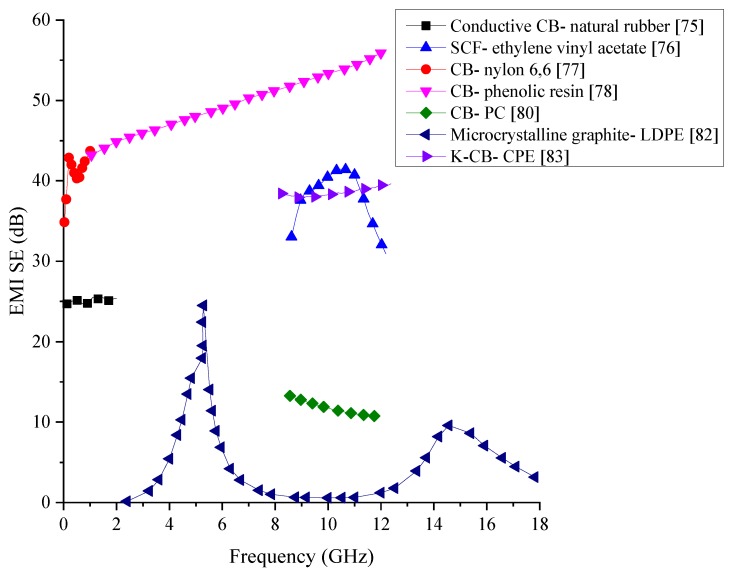
EMI SE comparison of reviewed polymer composites containing carbon black.

Critical data of the carbon black added EMI shielding polymer composite discussed in this section are provided in Table 2 Comparison of the polymer composites containing carbon black is graphically shown in Figure 4. Most of the composites reviewed within this section managed to generate a SE below 40 dB. However, the composite consisting of carbon black and phenolic resin has the highest SE between 45 to 55 dB with the SE increasing with the frequency. Most of the composite that has been fabricated has been tested only on a narrow range of frequency, making detailed comparisons between each other difficult. Since many of the composites have used the same type of CB particles, the variation of the SE could arise from the distribution and the type of matrix material used. Previous research conducted on EMI shielding materials have shown that materials with high conductivity have high SE in general. Since polymers are natural insulators, increasing the conductivity of the composite could increase the overall SE. While the addition of CB particles may help to increase the electrical conductivity of the composite, these particles alone would not be sufficient to create an excellent conducting network, which is essential for the EMI shielding. Therefore, the addition of another filler that might extend the conducting network would help to increase the SE of these composites.

### 3.3. Graphene-Based Polymer Composites

**Figure 5:**
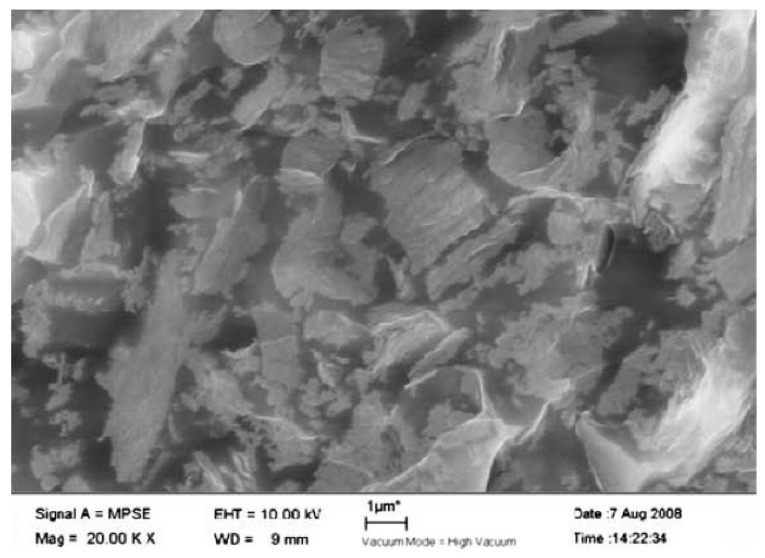
SEM image of the microstructure of graphene/epoxy composite. Reproduced with permission from Liang et al. [84], Copyright 2009, Elsevier Ltd.

Graphene and graphene oxide are also commonly used filler materials in polymer composite manufacturing since they can impart excellent electrically conductive properties to the composite. Graphene is known to be a single layer of carbon atoms arranged in the form of the hexagon, while graphene oxide is a product obtained through oxidising graphite [83]. Sheets of composite materials made with graphene mixed with epoxy have been tested for EMI shielding within 8.2 to 12.4 GHz frequency range. The morphology studies conducted through SEM images given in Figure 5, shows the proper distribution of graphene within the epoxy matrix. The electrical conductivity of the composite has shown to increase and attain a steady value after a specific amount of graphene. SE of the composite also shows an increase with the addition of graphene and a maximum value of about 21 dB has been obtained for the mix containing 15 wt% graphene [84].

**Figure 6:**
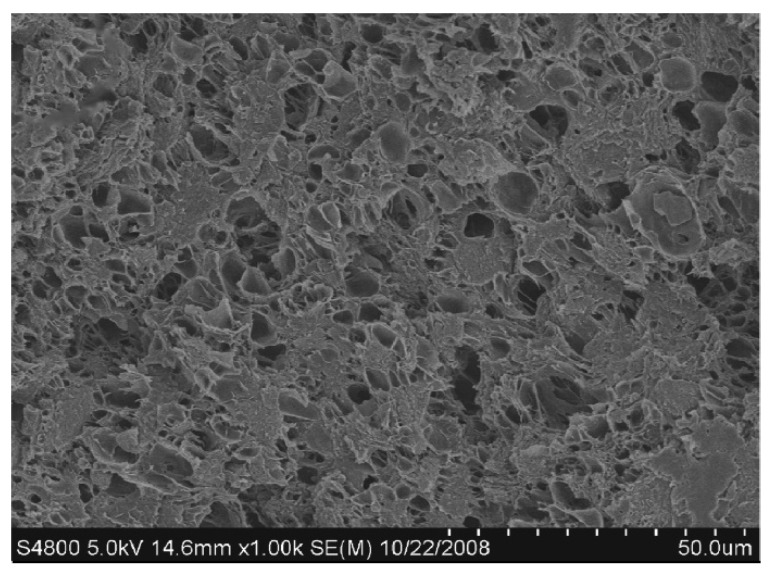
SEM image of the graphene/PMMA composite with microcellular cells. Reproduced with permission from Zhang et al. [85], Copyright 2011, American Chemical Society.

To improve the SE of graphene/polymer composites, foam material consisting of graphene as the filler and polymethylmethacrylate (PMMA) as the matrix has been fabricated with the aid of CO_2_ to form the microcellular cells. SEM image of the fabricated composite with microcellular cells is shown in Figure 6. The electrical conductivity of the composite has seen an increase due to the addition of graphene and has reached a value just above 10^−1^ S/cm when the graphene content is 1 vol%, and further addition of graphene has not increased the conductivity by a considerable amount. The tensile strength and the modulus of elasticity of the foam composite have reduced due to the presence of foam structure within the composite. The EMI SE of the composite has shown a steady increase with the addition of graphene. EMI SE range of 13 to 19 dB has been observed over the frequency range of 8 to 12 GHz in the foam composite containing 1.8 vol% of graphene [85]. The findings of this study show that it is possible to fabricate a composite containing graphene, which is tough and light in weight while having good EMI shielding properties.

**Figure 7:**
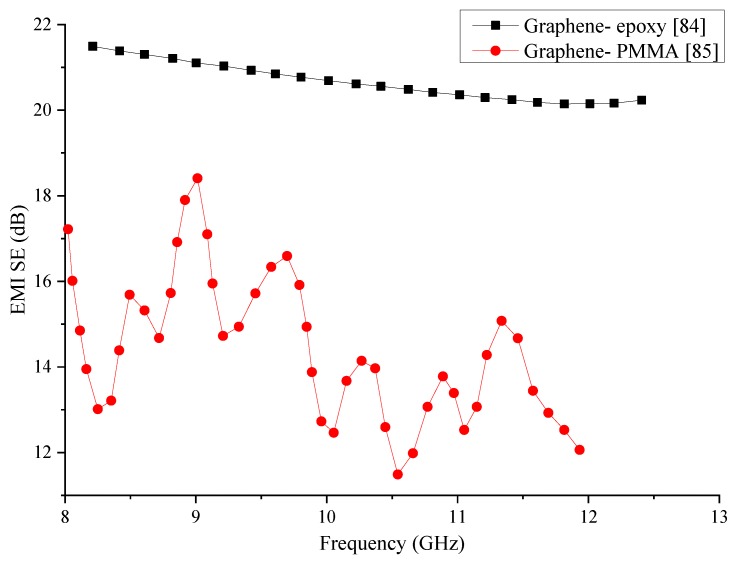
EMI SE comparison of reviewed polymer composites containing graphene.

Data of the two polymer composites containing graphene is tabulated in Table 3, while the SE of the two composites is compared graphically in Figure 7. The two composites show a drastic difference in their EMI SE within the same frequency range. While the epoxy matrix composite has been able to produce better and stable SE, the PMMA matrix composite has shown substantial variations in its SE. Regardless of the average SE produced by the PMMA matrix composite being lower, its fluctuation of the SE requires additional research to improve and stabilise its SE. The epoxy matrix composite has been able to generate better SE, but its SE has decreased slightly with the frequency. Even though PMMA is known to have higher electrical conductivity than most of the polymers, the PMMA matrix composite has produced a lower SE in this comparison.

### 3.4. Polymer Foams

Foam composite consisting of polyetherimide (PEI) matrix and graphene filler has shown improved SE compared to other polymer-based composites containing graphene. The creation of foam by using CO_2_ has been able to increase the SE from 17 dB to about 44 dB maximum value over the frequency range of 8 to 12 GHz. Apart from the increased SE, this composite is known to be exceptionally light in weight due to the inclusion of pores within the material and shown improved tensile and thermal insulation properties [86]. 

**Figure 8:**
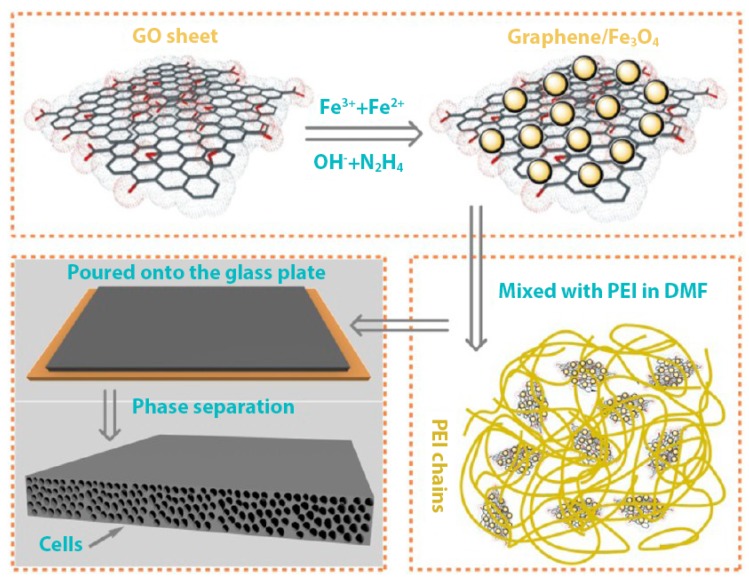
Process flow chart of preparing Fe_3_O_4_ coated graphene/PEI foam composite. Reproduced with permission from Shen et al. [87], Copyright 2013, American Chemical Society.

To further increase the SE of graphene mixed polymer composites, Fe_3_O_4_ particles deposited graphene sheets has been utilised in creating a foam composite. The matrix of this composite is made of polyetherimide (PEI). The resultant composite has shown excellent flexibility and light weight properties. The process followed in preparing this composite is shown graphically in Figure 8. The addition of Fe_3_O_4_ particles to the graphene has increased the SE of the overall composite dramatically compared with other composite synthesised with graphene for EMI shielding applications. The composite containing just 10 wt% filler has shown a SE of about 41.5 dB over the frequency range of 8 to 12 GHz. In addition to the increased SE, this composite has also shown improved paramagnetic properties and thermal conductivity due to the inclusion of Fe_3_O_4_ particles [87].

**Figure 9:**
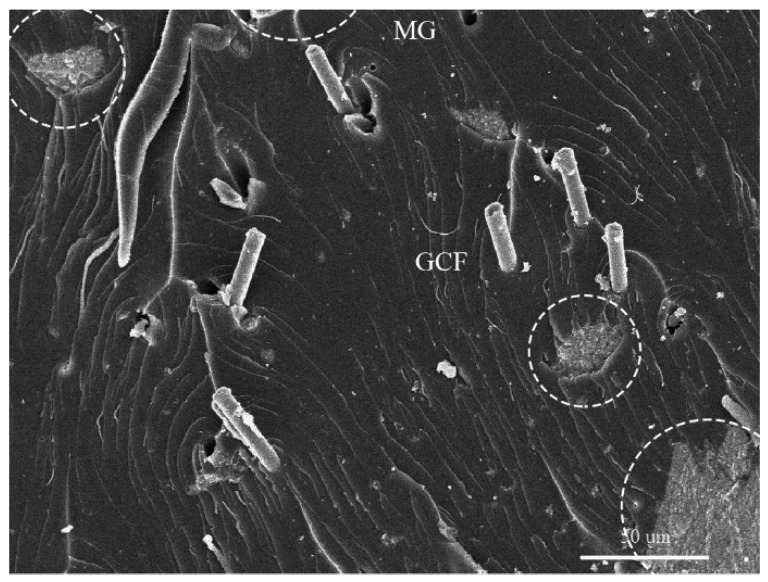
microstructure of reduced graphene oxide (rGO) coated with carbon nanofibers and Fe_3_O_4_ nanoparticles deposited rGO. Reproduced with permission from Wu et al. [89], Copyright 2017, Elsevier Ltd.

**Figure 10:**
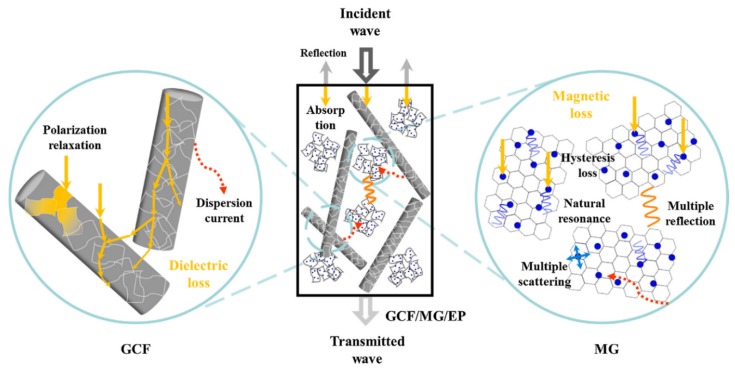
Illustration of a possible interaction between EMW and composite with epoxy matrix and reduced graphene oxide (rGO) coated with carbon nanofibers, and Fe_3_O_4_ nanoparticles deposited rGO nanohybrids. Reproduced with permission from Wu et al. [89], Copyright 2017, Elsevier Ltd.

Composites with an epoxy matrix and reduced graphene oxide (rGO) coated with carbon nanofibers (GCF) and Fe_3_O_4_ nanoparticles deposited rGO nanohybrids (magnetic graphene- MG) have been tested for the SE within 8.2 to 26.5 GHz frequency range. rGo is the product obtained after converting graphene oxide to pristine graphene [88]. The SEM image of the synthesised composite shown in Figure 9, shows the distribution of each filler within the epoxy matrix. The shielding test has shown a SE of 31.3 dB to 51.1 dB, which is a high value for a polymer composite containing graphene. Possible reasons for the high SE can be attributed to the dielectric and magnetic loss imparted by each component within the composite, as illustrated in Figure 10 [89].

**Figure 11:**
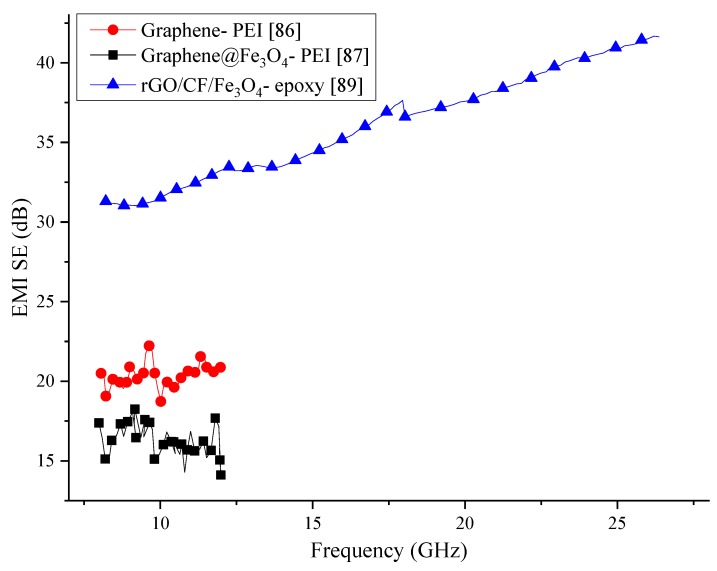
EMI SE comparison of reviewed foam polymer composites.

Most of the research on graphene has shown that it has considerably good electrical conductivity, hence the use of graphene in the fabrication of the EMI shielding composite has been on the increase. Information of the polymer foams reviewed in this section is provided in Table 4. From the comparison of the composites reviewed in this section, which is graphically represented in Figure 11, it can be concluded that the addition of graphene to polymers can enhance the SE. However, the SE generated by the composite containing reduced graphene oxide has shown a remarkably high SE compared to other composites.

Additionally, the SE generated by the composite containing rGO has been stable over a wide range of frequency, making it ideal to be used in a wide variety of applications. The high SE of rGO/polymer composite can be attributed to the high electrical conductivity that arises as a result of reducing graphene oxide back into graphene form. The stable and high SE can also be produced due to the formation of an excellent conducting network and the absorption of EMWs from the ferrite particles. Many studies in the fabrication of EMI shielding composites have shown that in order to achieve high SE, multiple fillers needed to be added, which have a synergetic effect in enhancing the SE. The results obtained from the rGO/polymer composite within this section conform to these findings.

### 3.5. Carbon Fibre-Based Polymers 

Carbon fibres (CF) have been investigated for their excellent conductivity and high strength as a filler material for polymer composites for a very long time. Due to the high conductivity of CF, composites that are synthesised are known to have good EMI shielding properties as well. Nylon-6,6 matrix composite containing carbon fibres of different lengths have tested for SE in order to assess how the length and the weight fraction of the fibres affect the SE of the composite. Results from testing have shown that better SE can be achieved with long CF than short ones. While composites with short CF have not shown a significant change in EMI SE with the frequency, composites with long CF have shown an increase in SE with the frequency. The electrical conductivity of the composite has also increased with CF percentage, especially in the matrix containing long CFs. The overall SE in the tested frequency range of 1 to 1500 MHz has varied between 45 to 75 dB at 30 wt% for long CF composite while for short CF composite with the same loading has yielded only a SE of about 30 dB [90]. The experiment has shown that the SE of this composite depends both on the concentration and length of the CFs. 

The orientation and the weight percentage of CF in liquid crystal polymer composites have been investigated over the frequency range of 1 to 1500 MHz. Results from the shielding tests have shown that a random orientation of CF is not going to create a composite with good shielding properties. Since longitudinal fibres, when oriented parallel to the electric field would reflect a higher percentage of EMWs, the parallel fibre embedded composites have shown higher SE. These composites have shown a maximum SE of about 50 to 60 dB in the tested frequency range. Comparison with the composite containing nylon-6,6 matrix, liquid crystal polymer matrix composite has shown superior shielding properties [91].

To improve the EMI SE of CF-added composite materials, activated CF has been mixed with epoxy matrix and tested for its SE. CF used in the fabrication of this specific composite has been activated by using CO_2_ after heating to 1000 °C in a N_2_ atmosphere. The testing frequency range has been from 1.0 to 1.5 GHz. In this frequency range, the composite has shown a SE of about 39 dB. The increased SE of the composite attributes to the increased multiple reflections of EMWs when CFs are activated. Apart from the increased SE compared to conventional CF used composites, the tensile strength of the composite has also shown improvement due to the increase of the tensile strength of CF after activation [92]. In similar research, polyamide resin matrix composite fabricated with activated carbon fibres using steam has been tested for the shielding properties in the frequency range of 2 to 18 GHz. Results have shown that this composite can have a reflection loss below 10 dB with a maximum of 28 dB [93].

**Figure 12:**
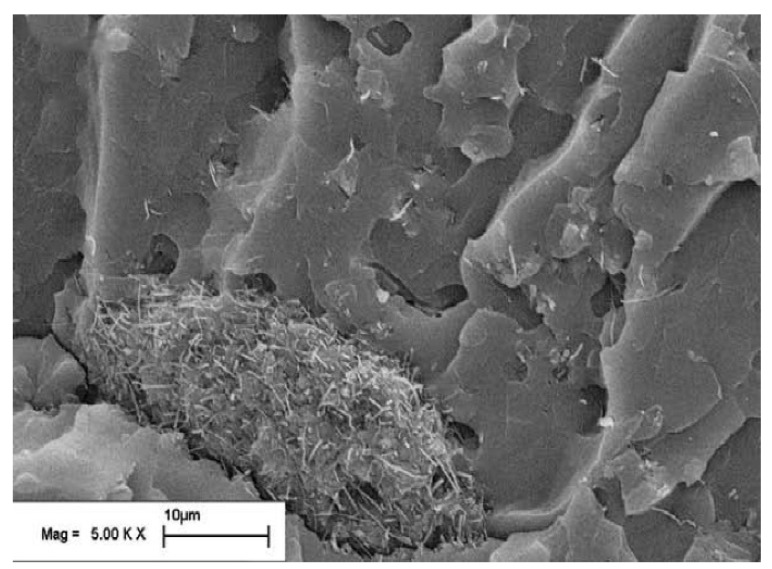
SEM Image of microstructure with carbon nanofiber aggregate within polymer matrix. Reproduced with permission from Nanni et al. [94], Copyright 2009, Elsevier Ltd.

To investigate the effect of dispersion method on the SE of CF reinforced polymer composites, two dispersion methods using evaporation and filtration for the removal of organic solvent have been used. The microstructures of the resultant composites have been analysed using SEMs, which shows that the solvent evaporation technique has much better dispersion than the filtration technique. CF has seen to get agglomerated in the microstructure, as shown in Figure 12 when the filtration process used in the fabrication process. Due to the better dispersion of CFs in the evaporation method, the composite has also shown better electrical conductivity and shielding properties. The prepared composites have been tested for their SE in the frequency range of 8 to 20 GHz. Within this frequency range, the composite synthesized using the evaporation method has shown a maximum absorption peak of 18.3 dB with 4 wt% CF loading while the filtration based composite has achieved only a 10.8 dB absorption peak with the same CF loading. This observation leads to the conclusion that the dispersion method has a significant impact on the shielding properties of carbon fibre reinforced composites [94].

With the development of nanotechnology and the use of carbon nanofibers, there has been a rapid growth in the different type of composite materials used for different applications, including in EMI shielding. Carbon nanofibers (CNF) with a diameter between 50 to 150 nm have been used to create a composite with a matrix of polyacrylonitrile (PAN)/polymethyl methacrylate (PMMA) (mass ratio of 3/7) for the shielding purpose. The EMI SE of the composite has been tested in the frequency range of 8.2 to 12.4 GHz. A composite containing 8 wt% CNF has shown a peak in EMI SE of 34 dB at 10.5 GHz. Composites with other compositions of CNFs have not shown such high peaks of EMI SE within the frequency range tested. In addition to the increase of the SE, the increase of CF content has also increased the permittivity and the permeability of the composite [95].

Since the presence of microspheres within the composite has shown an increase in the shielding properties in many research, the same method has been utilised to fabricate a composite with CNF as the reinforcement, phenolic resin as the matrix, and hollow carbon microspheres (HCMs) to create the foam structure. The resultant composite has been tested within the frequency range of 300 kHz to 8 GHz. To achieve high fracture toughness, the HCM content was limited to 28 vol%. Different percentages of CNF have been added to find out the optimum content for best SE. It has been found that with the increase of CNF content, the SE also increases. The SE of the composite containing 2 vol% CNF is within 20 to 25 dB range. The addition of HCMs has increased the SE by expanding the conducting network but also has negatively impacted it by creating large surfaces within the composite. Results also indicate that the SE obtained in this composite are better than most CF reinforced polymer composites [96]. 

Composite fabricated with a CNF and polysulfone has been investigated for the optimal CNF content for EMI SE. Synthesised composite has been tested within 8.2 to 12.4 GHz frequency range and yielded a SE of about 45 dB for 10 wt% CNF loading. SEM analysis of the microstructure has revealed the CNFs have been distributed evenly within the matrix, which has helped to create an excellent conducting network and increase the EMI SE. Since the thickness of the tested composite is only 1 mm, the use of this composite shows promising results for future developments [97].

**Figure 13:**
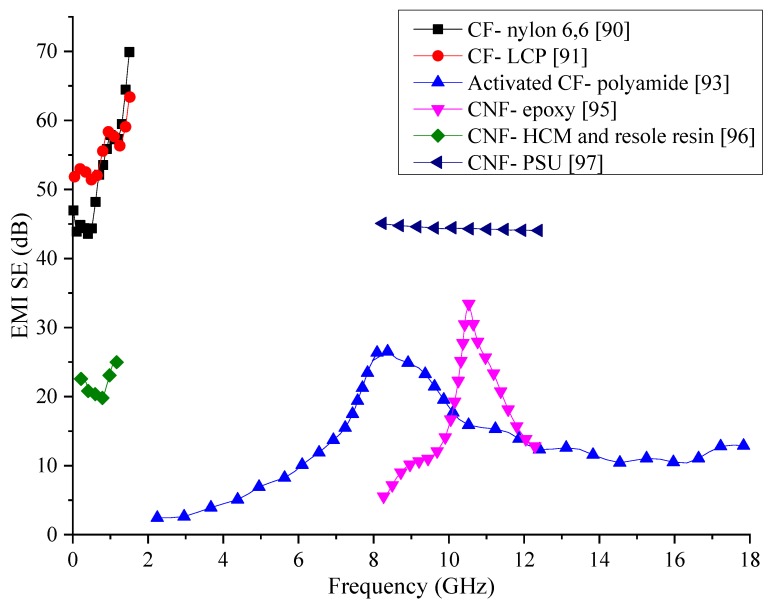
EMI SE comparison of reviewed polymer composites containing carbon fibres.

Carbon fibre has long been used an excellent conductive filler even though there are better conductive fillers, the lower cost of the CF that has resulted from developed manufacturing processes has made it popular in composite fabrication. Information of composites reviewed within this section are tabulated in Table 5 and are graphically compared in Figure 13. The results are difficult to be compared since the composite specimens have been tested in different frequency ranges. However, many of the composites in the comparison show varying SE values. The composite consisting of CFs in PSU matrix has been able to generate rather constant SE within the tested frequency range. Composites with CF/nylon-6,6 and CF/LCP has shown higher SE at lower frequencies, but their SE has shown a significant variation with the frequency. More research would be necessary to stabilise the SE produced by these two composites if they are to be used in practical applications. Other composites with CFs have shown lower SE, which may require the addition of multiple fillers along with CFs to enhance the SE to required values.

### 3.6. Single-Walled Carbon Nanotubes Based Polymers

Carbon nanotubes (CNT) are also a widely used filler in polymer composite fabrication due to their high conductivity. A test using SWCNT in epoxy resin has been conducted to analyse the effect of length of the nanotubes on the SE and the conductivity of the composite. The SE has been tested in the frequency range of 10 MHz to 1.5 GHz. To make sure proper dispersion of the CNTs in the matrix, several sonication steps have been followed during the syncretization of the composite. The results have shown that with the increase of the CNT, the SE and the electrical conductivity of the composite increase. Composite with the longer CNTs has shown better shielding and conductive properties with a maximum SE of 49 dB at 10 MHz. The overall SE has been 15 to 20 dB within the frequency range of 500 MHz to 1.5 GHz [98]. The EMI SE of the composite has shown a gradual drop with the increase of the frequency. The results from this test have shown that SWCNT can be used in the fabrication of a lightweight composite for EMI shielding applications. 

To study the EMI SE of SWCNT-added polymer composites in higher frequencies, a composite consisting of SWCNT and polyurethane had been fabricated and tested in the frequency range of 8.2 to 12.4 GHz. Results from this test have also shown that the SE of the composite increases with the increase of SWCNTs content and the SE and electrical conductivity correlate with each other. The composite has yielded an overall SE of about 17 dB for the tested frequency range. The dominant shielding mechanism has shown to be the absorption of the EMWs, and with the increase of the CNTs, the reflection mechanism has shown an increase [99]. In another research, composites consisting of SWCNT/polymer, MWCNT/polymer, and carbon foam composites have been tested for their EMI SE in the frequency range of 26.5 to 40 GHz to assess the filler with best shielding properties. Two forms of SWCNTs have been used in this study, one which is the arc discharged CNTs, and the other is commercially available CNTs made by a chemical vapour deposition method. SE results for these composites have shown that the addition of individual SWCNTs does not increase the SE by a significant degree. MWCNTs, on the other hand, have shown to be an excellent additive to increase the SE of the polymer composite. However, composite made using bundles of SWCNTs has shown better SE than the composite with MWCNTs and known to be more economical. However, out of all the composites fabricated and tested, the carbon foam has shown a high SE compared to other composites. Apart from high SE, the density of the foam composite has also been the lowest [100].

**Figure 14:**
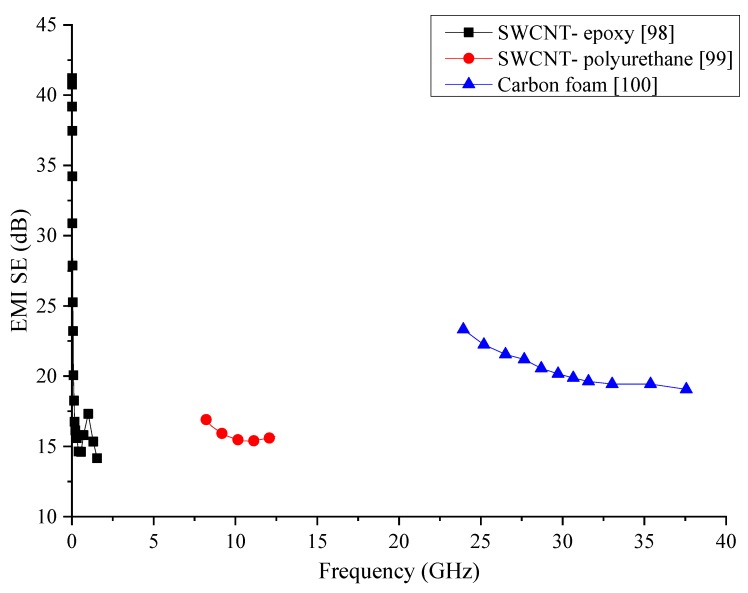
EMI SE comparison of reviewed polymer composites containing SWCNTs.

Carbon nanotubes have revolutionised the composite industry with their high electrical conductivity and enhanced mechanical properties. Since their discovery, the manufacturing processes of CNTs have undergone many developments which have reduced the manufacturing cost while enhancing their properties. Data of polymeric composites consisting of SWCNTs reviewed in this section are summarised in Table 6 and graphically compared in Figure 14. Almost all the composites containing SWCNTs has shown a SE about 15 to 20 dB in their respective frequency range they have been tested. Moreover, the SE produced by these composites has shown a decrease with the increasing frequency. 

The SE produced by SWCNT/epoxy has shown a significant instability with the SE dropping drastically with the frequency. The carbon foam composite fabricated to compare the SE with other composites is the only one in this comparison that has been able to produce stable and considerably high SE. However, even its SE is only about 20 to 25 dB, which may require enhancement to meet industry requirements.

### 3.7. Multi-Walled Carbon Nanotube-Based Polymers

MWCNTs have been investigated in many conductive and EMI shielding materials research due to their extremely high electrical conductivity. The concentric multiple tube structure of MWCNTs are the main reason for these reinforcements having higher conductivity. With the inclusion of MWCNTs, polymer matrix composites are expected to increase their overall conductivity and improve the EMI SE. MWCNTs made by catalytic decomposition of a ferrocene-xylene mixture in a quartz tube reactor have been used in one of the research to create an EMI shielding composite by mixing with poly(vinylidene fluoride) (PVdF) and poly(vinyl pyrrolidone) (PVP) polymers. The measurements of the electrical conductivity of the composites have shown that there is a rise in the conductivity with the MWCNT content, and it reaches a saturated value after about 0.4 wt% of MWCNTs content. The synthesised composite has shown an EMI SE of 18 to 21 dB in the frequency range of 10 to 1500 MHz, which is a significantly high value when compared with a composite containing the same percentage of carbon fibres. Annealing of the composite has also been investigated to find the effect of the process since it is known to increase the SE of carbon fibre reinforced composites, but have proven to lower the SE it in this instance [101].

In the hope of fabricating a lightweight EMI shielding composite, MWCNT/polystyrene (PS) foam composite has been fabricated and tested in the frequency range of 8 to 12 GHz. With a loading of 7 wt% of MWCNTs, the composite has demonstrated a SE of about 20 dB. Even though this is not an extremely high SE value, the composite has demonstrated a low density and higher EMI SE than CF reinforced polymer composites, showing promise for future developments [102]. In another research composite consisting of MWCNTs and polycaprolactone (PCL) matrix has been fabricated in two different forms (solid and foam) to investigate the SE within 40 MHz to 40 GHz frequency range. The foam composite consisting only 0.25 wt% of MWCNTs has shown excellent shielding properties of 60 to 80 dB within a 25 to 40 GHz range which is significantly higher than the solid composite containing a higher fraction of MWCNTs. SEM analysis has shown the microstructure consisting of pores with well-distributed MWCNTs in these composites. Even though reflection has been the primary shielding mechanism in the solid composite, absorbance has been the primary shielding mechanism in the foam. The higher SE of the foam composite can be attributed to the effect of both MWCNTs and foam within the composite acting to reflect and absorb the EMWs [103]. The high SE, even at lower reinforcement content, has shown that MWCNTs can be used effectively in creating a good EMW shielding composite.

MWCNTs made by chemical vapour deposition have been used to fabricate composites with polymethyl methacrylate (PMMA) and polystyrene (PS) as the matrix material for shielding against EMI using the solvent casting method. The fabricated composites have been tested within the frequency range of 8.0 to 12 GHz for their SE, and an average value of 18 dB has been obtained as the SE for both composites. In both types of composites, the electrical conductivity had seen an increase when the MWCNT content was increased and has reached a saturation value. SEM analysis of the fracture surface of composites has shown even distribution of the nanotubes within the matrix. The addition of the nanotubes has shown an increase in the mechanical properties of the composite as well. Overall the SE displayed by this composite has demonstrated that even though it is not an extremely high value, it is enough for these composites to be used in small electronic applications due to their lightweight [104].

**Figure 15:**
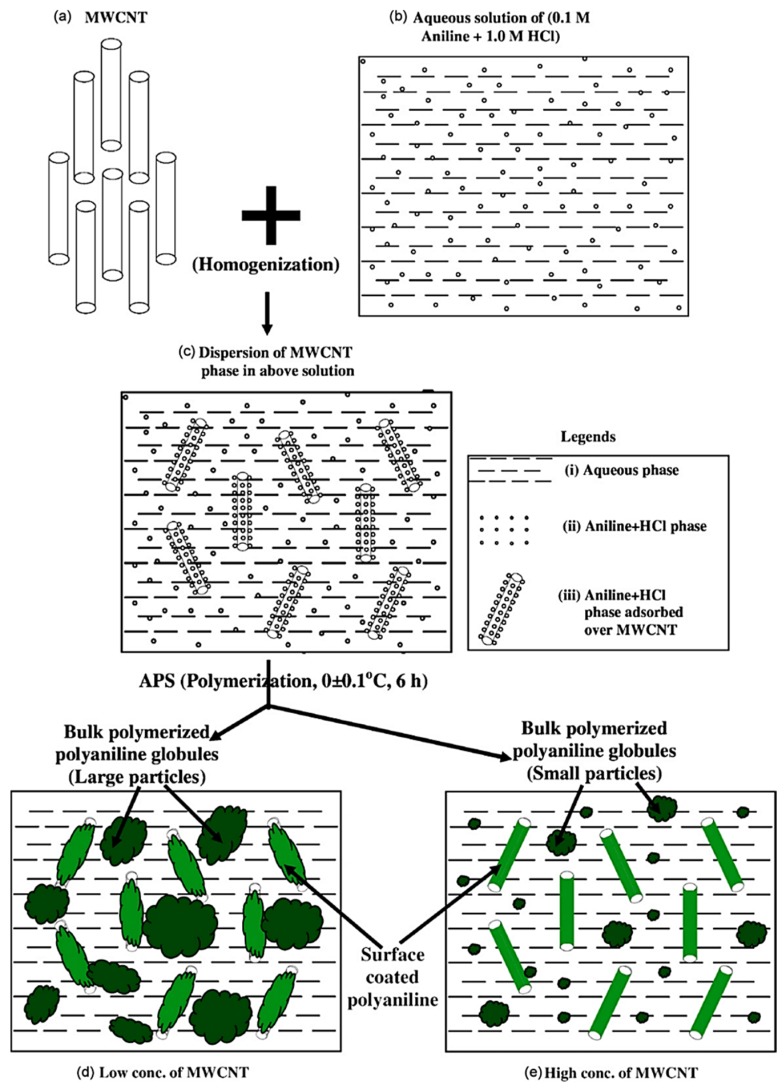
Graphical representation of the formation of PANI–MWCNT nanocomposites. Reproduced with permission from Saini et al. [105], Copyright 2009, Elsevier Ltd.

**Figure 16:**
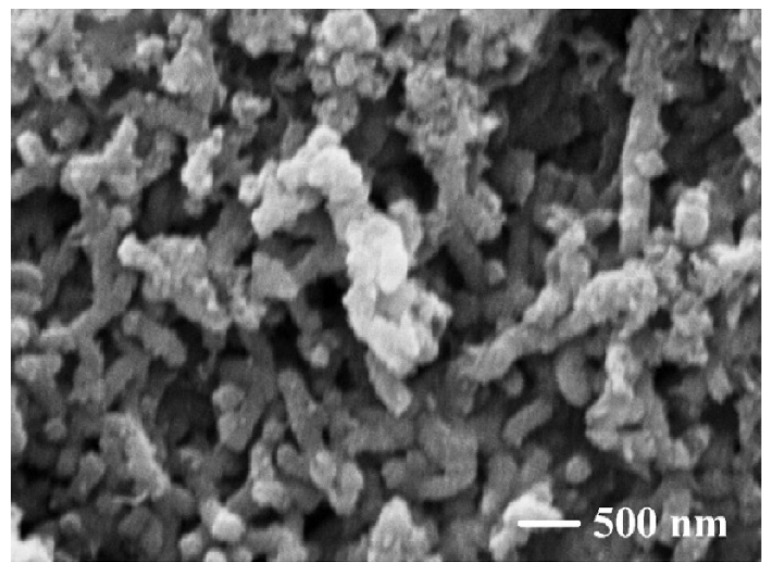
SEM image of composite containing MWCNTs in PANI matrix. Reproduced with permission from Saini et al. [105], Copyright 2009, Elsevier Ltd.

Due to its inherent conductivity, PANI has been studied as a potential matrix material in many of EMI shielding polymer composite studies. However, since the conductivity of PANI is not as high as that of metals, additional reinforcements are needed to achieve an adequate level of SE. MWCNTs have been investigated as potential reinforcement in PANI based polymer composite in the frequency range of 12.4 to 18.0 GHz. Synthetization of the composite has been carried out similar to that of the preparation of pure PANI from aqueous solution but with the addition of MWCNTs as shown in Figure 15. After the preparation of the composites with various percentages of MWCNTs, they have been subject to various characterisation tests. The addition of MWCNTs has shown to increase the conductivity of the composite higher than either individual PANI or MWCNTs. The synergistic effect of the two phases in the composite would have been the reason for the composite having such a high conductivity than the filler or the matrix. SEM micrograph is shown in Figure 16 indicates the distribution of MWCNTs within the matrix. As expected, the composite also shows good SE of 27.5 to 39.2 dB within the tested frequency range with the primary shielding mechanism being the absorption [105].

In an attempt to increase the SE of MWCNT epoxy composites, fluorination of the nanotubes has been carried out to improve the dispersion and the adhesion of the MWCNTs to the matrix. The improved distribution of the nanotubes has been confirmed by the UV spectra carried out on the specimens. Fluorination of the MWCNTs has increased the SE of the composite, and an average value of 28 dB has been achieved for the frequency range of 1 to 4 GHz. Along with the SE, permittivity and permeability have also increased due to the process above [106].

Two main reasons why some of the composites reinforced with MWCNTs achieve poorer SE than expected are the poor dispersion and interfacial interaction with the matrix and MWCNTs. Mechanical dispersion is a commonly used approach to overcome these problems by having a thorough distribution of the nanotubes within the matrix. However, the dispersion achieved by this method is still insufficient to create a very good distribution of the nanotubes within the matrix [107]. However, a process known as functionalization, which introduces side groups to the walls of the nanotubes, would help to disperse them within the matrix properly while enabling them to adhere to the matrix strongly [108].

Functionalization of MWCNTs can be carried out in many different chemical routes. SE of such functionalized MWCNTs and polymethyl methacrylate (PMMA) composite has been studied in the frequency range of 2 to 18 GHz. Characterisation methods, including SEM, have revealed that MWCNTs have indeed been distributed adequately within the polymer matrix. The composite has been able to generate a SE of 13 to 18 dB with a 1 mm thick specimen containing 4.76 wt% loading of MWCNTs. Electrical conductivity and the SE both have shown an increase with the increase of the MWCNTs loadings. However, there has been no correlation between the conductivity and the SE that the researchers were able to identify [109]. 

**Figure 17:**
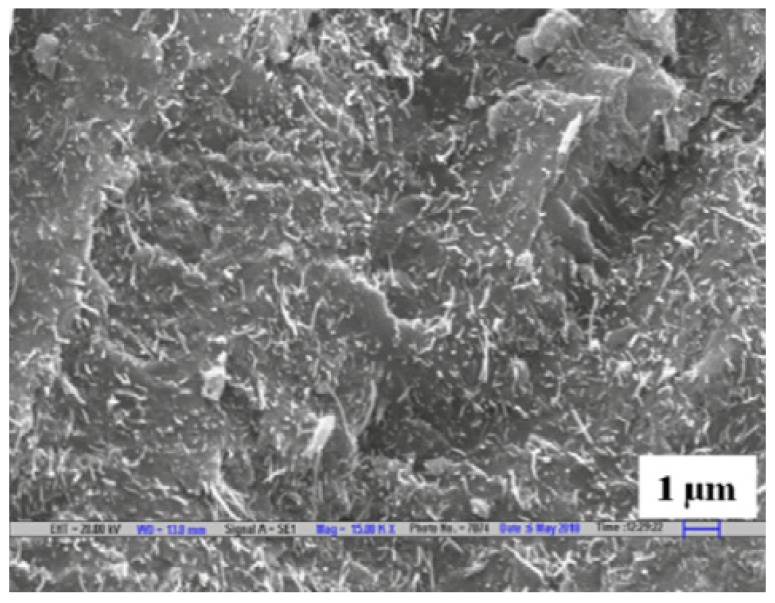
SEM image of MWCNTs distribution within PTT matrix. Reproduced with permission from Gupta et al. [110], Copyright 2011, Elsevier Ltd.

The EMI SE of a composite consisting of various percentages of MWCNTs and poly(trimethylene terephthalate) (PTT) has been investigated in the frequency range of 12.4 to 18 GHz. SEM micrographs, shown in Figure 17, has shown that MWCNTs have been distributed throughout the PPT matrix. With the increase of the nanotube content, both SE and the electrical conductivity have increased. While the conductivity has reached a saturation value with the increasing nanotube content, the SE has shown a linear increase. Composite with 10 wt% MWCNT loading has shown a SE of 36 to 42 dB in the measured frequency range and shown an increase with the frequency. Absorption has been identified as the primary shielding mechanism in this composite [110]. The same combination of reinforcement and matrix has been tested for its SE in the frequency range of 8.2 to 12.4 GHz in subsequent research but with lower loadings of MWCNTs. SE achieved for this frequency range has been about 23 dB but with only 4.76 vol% of nanotubes loading. In this instance, also both the conductivity and the SE have shown an increase with the MWCNT content [111].

The effect of orientation of MWCNTs within the matrix has been researched by formulating a composite with a polycarbonate (PC) matrix. The nanotubes have been distributed within the matrix, and then the composite has been injected into a dog bone mould to study its various properties. Both the electrical conductivity and the SE has shown higher values in the direction parallel to the nanotube orientation since the electrical network can be theorised to be more interconnected in this direction. A maximum SE of about 25 dB in the frequency range of 8.2 to 12.4 GHz has been obtained in these composites with 15 wt% MWCNT loading [112]. A comparative study on composites fabricated for EMI shielding applications has been carried out by synthesising injection moulded, and compression moulded samples of MWCNT/polystyrene (PS) composites. Injection moulded specimens are expected to have a specific orientation of the nanotubes, which is along the direction of the injection, and compression moulded specimens are expected to have a random orientation of the nanotubes. Upon testing these composites with various filler loadings for their electrical conductivity and SE, it has been found that random orientation of the fibres is preferred over a specific direction. For composites with 20 wt% of MWCNTs the injection moulded composite has shown a SE of about 45 dB while for the same loading compression moulded composite has shown a SE of about 55 to 65 dB within the same frequency range. Electrical conductivity and permittivity of the specimens also show a higher value for the compression moulded specimens. This indicates that the random orientation of the nanotubes results in a better-conducting network within the composite which leads to higher SE even though authors have not found a direct correlation with the conductivity and the SE [113].

To assess the properties of MWCNTs/polymer composites at higher frequencies, polyhedral oligomeric silsesquioxane (POSS) matrix-based composite has been synthesised by grafting the polymer to the nanotubes. MWCNTs have been dispersed within the matrix homogeneously. The composite has been tested for EMI shielding in the frequency range of 36 to 50 GHz. As with other MWCNT reinforced composites, the electrical conductivity has increased with the filler loading and has achieved a saturation value. The composite with only 4 wt% loading of nanotubes, has displayed a SE of 15 to 16 dB in the tested frequency range and the SE has increased slightly with the frequency. This is a comparatively a good shielding value for a composite with low filler content and at high frequencies, which has resulted due to the grafting of the polymer to the nanotubes [114].

**Figure 18:**
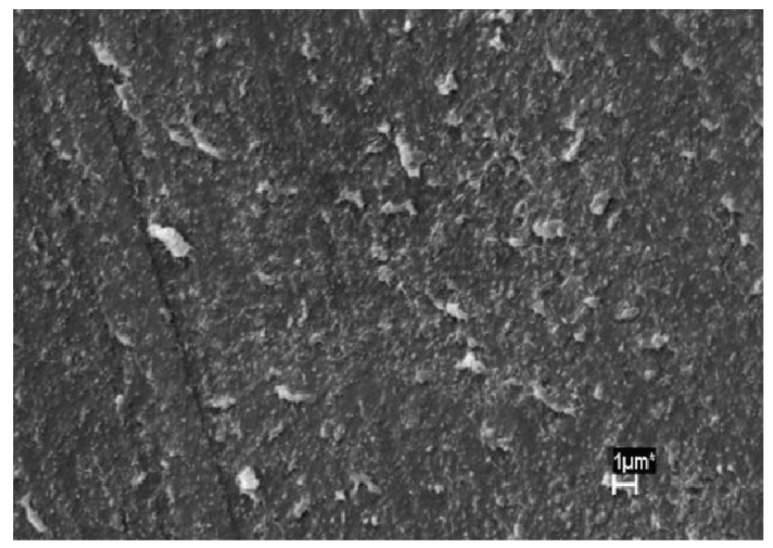
SEM image of the coating consisting of MWCNTs in polyurethane matrix. Reproduced with permission from Folgueras et al. [116], Copyright 2014, Scielo.

Coatings that can be used for EMI shielding have also been of great interest since a coating that can be applied over an existing structure could eliminate the need to fabricate the entire structure, saving time and money. Since many existing coatings are polymer-based, the newly studied coatings are also polymer-based composites with reinforcements acting to either absorb or reflect the EMWs. A coating of MWCNTs/PMMA has been researched for this reason in the frequency range of 100 MHz to 14 GHz. The composite consisting of 25 wt% of MWCNTs, has been fabricated by dispersing the nanotubes within the epoxy by using ultrasonication. The resultant has been made into thin films of about 100 μm in thickness after ball-milling the initial product. The shielding test results have yielded a SE above 20 dB for the frequency range, which is a considerably high value considering the thickness of the specimen [115]. In similar research, a coating made with MWCNTs dispersed within a polyurethane matrix has been applied onto a laminated composite made with polyphenylene sulfide and glass fibre and tested for SE within 8 to 12 GHz frequency range. However, in this research, not only the coating but also the backing material has acted as an EMI shield, blocking about 99% of the waves being transmitted through the entire material. SEM analysis of the coating given in Figure 18 shows the distribution of MWCNTs within the matrix. Reflection of EMWs from the coating has seen a reduction with the increase of the frequency [116].

**Figure 19:**
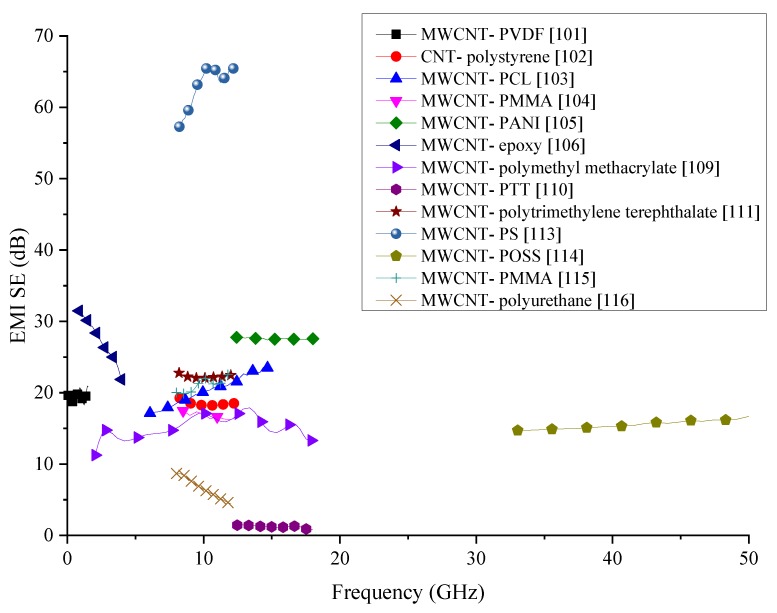
EMI SE comparison of reviewed polymer composites containing MWCNTs.

Even though MWCNTs are renowned for their very high electrical conductivity, their high manufacturing cost is one of the significant drawbacks that limit their applications to research uses. With high electrical conductivities, MWCNTs are ideal fillers to be used for the fabrication of EMI shielding composites. Information about MWCNT-incorporated polymer composites reviewed in this section are summarised in Table 7, and the comparison of the SE produced by the composites with MWCNTs reviewed in this section is shown in Figure 19. Most of the composites analysed show a SE between 15 and 25 dB. While PTT and Polyurethane matrix composites have shown even lower SE than this range, PS matrix composite has been able to produce the highest SE in this group. For the fabrication of the MWCNTs/PS composite authors have used compression moulding technique, which has remarkably high SE than injected moulded specimens in the same research. The main reason for the high SE put forth by the authors is the random distribution of the CNTs within the denser matrix. The 3D distribution of the CNTs could have increased the overall SE by extending the electrically conducting network within the composite. While MWCNTs boast of high electrical conductivity, their proper distribution is also critical for achieving high EMI shielding properties. As an additional step, the effect of multiple fillers with a combination of MWCNTs could be explored for the fabrication of composites with even higher EMI SE.

### 3.8. Polymer Composites with MWCNTs and Mixed Fillers

**Figure 20:**
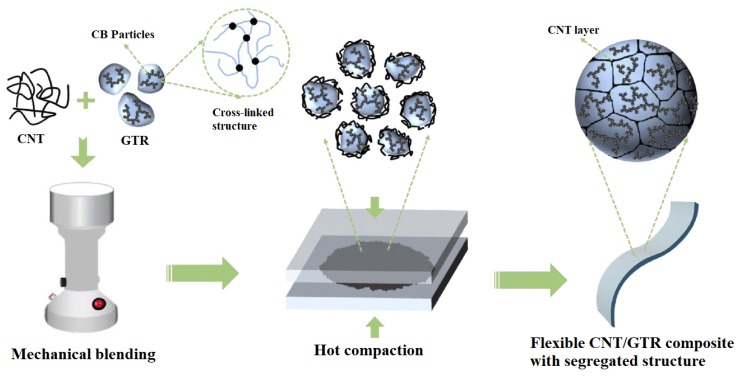
Schematic representation for the fabrication of flexible MWCNT/GTR composite. Reproduced with permission from Chuan et al. [118], Copyright 2017, Elsevier Ltd.

**Figure 21:**
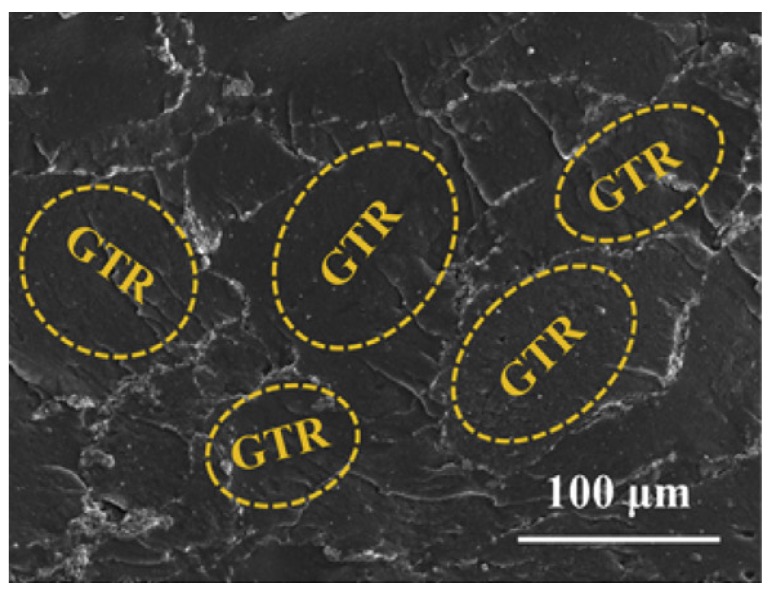
SEM image of the MWCNT/GTR composite. Reproduced with permission from Chuan et al. [118], Copyright 2017, Elsevier Ltd.

In many studies focusing on creating composites for EMI shielding, use of just one reinforcement has been shown to be insufficient to create the required SE resulting in a large amount of filler loading in the composite which has led to the eventual increased cost of the composite. Therefore, the use of more than one type of filler has been investigated by many researchers and have proven to be successful in increasing the overall SE of the composite in many instances. The same approach has been used in research where microscale silver flakes (Ag flakes) and multi-walled carbon nanotubes decorated with nanoscale silver particles (nAg-MWCNTs) have been used as fillers and nitrile butadiene rubber (NBR) as the matrix. One of the key advantages of this composite has been the flexibility of the composite owing to the rubber matrix. The fabricated composite has been tested for SE in 30 MHz to 1.5 GHz frequency range and its electrical conductivity. The results have confirmed that the addition of Ag nanoparticles on MWCNTs has increased not only the SE but also the electrical conductivity. The composite has shown a SE of above 40 dB in the tested frequency range. Authors have been able to identify a linear relationship between the logarithmic value of electrical conductivity and the SE of the material [117]. In a similar attempt to fabricate a flexible composite to shield against EMI, MWCNTs have been added to ground tire rubber (GTR). One key advantage of this research is the value added to the rubber tire wastes. The fabrication process followed by the researchers is represented schematically in Figure 20. SEM micrograph showing the GTR matrix and the MWCNTs is shown in Figure 21. With 5 wt% loading of MWCNTs, this composite has been able to achieve a SE of 60 to 70 dB within the frequency range of 8 to 12 GHz, which is a significantly high value for a specimen having a thickness of 2.6 mm. Even after many bending cycles of the flexible composite, it has been able to maintain the same SE, which pave the way for future researches in flexible composites for EMI shielding [118].

MWCNTs mixed in with gold (Au) nanoparticles in a PANI matrix form a polymer matrix composite with high EMI SE. Since all the elements that make up the composite are good conductors, their combined effect has been expected to increase the overall conductivity of the composite. Au nanoparticles have been added in two different ways; one is the direct mixing of Au nanoparticles with MWCNTs in PANI matrix and the second is coating the MWCNTs with AU nanoparticles and then polymerisation of PANI. Out of the two methods, the latter has proven to have better properties in terms of conductivity and SE. For the SE a frequency range of 8 to 12 GHz has been used. The composite with improved properties has shown a SE of 16 to 56 dB, and the absorption as the primary mechanism of shielding [119]. 

Fe_3_O_4_ is known to be a good filer material to be added to composites for EMI shielding since these particles can absorb the energy within the EMWs. A composite where Fe_3_O_4_ nanoparticles with grafted MWCNTs have been added to a matrix of PC (polycarbonate)/SAN [poly(styrene-co-acrylonitrile)] blend has been investigated for its EMI SE. The synthesised composite has been tested for SE in the frequency range of 8 to 18 GHz. SEM analysis has shown the dispersion of the two fillers within the polymer blend. Electrical conductivity test carried out for the specimens has also shown that the conductivity increases with the filler content and would reach an optimum value at a specific filler content, showing behaviour similar to most composites with MWCNTs. The composite has shown a SE of about 23 to 32 dB in the tested frequency range. Considering there was only 3 wt% of filler within the composite this has been a high value of SE when compared to values in literature, indicating the addition of multiple fillers could increase the overall SE of the composite [120].

**Figure 22:**
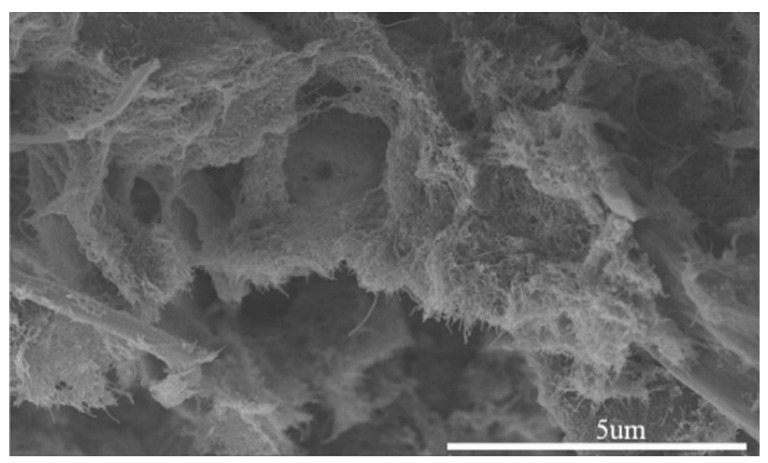
SEM image of the MWCNT/PVDF porous composite. Reproduced with permission from Wang et al. [121], Copyright 2016, Elsevier Ltd.

Creating a porous structure within the composite is another proven method in creating an EMI shielding material. A composite containing MWCNTs and a porous matrix of poly(vinylidene fluoride) (PVDF) has been tested for the SE within the frequency range of 8 to 12 GHz. The thickness of the tested specimen has been just 2 mm. The SEM images of the composite given in Figure 22, shows the porous structure of the composite with the MWCNTs embedded in the matrix. The creation of a 3-dimensional network of nanotubes within the composite as a result of introducing the pores has resulted in an increase in the conductivity and the EMI SE compared to other polymer matrix composites. The composite containing 15 wt% of MWCNTs has achieved an average SE of 56 dB in the tested frequency range. Another advantage this composite has over the other is the reduction of the density due to the porous structure, making it lighter in weight [121].

**Figure 23:**
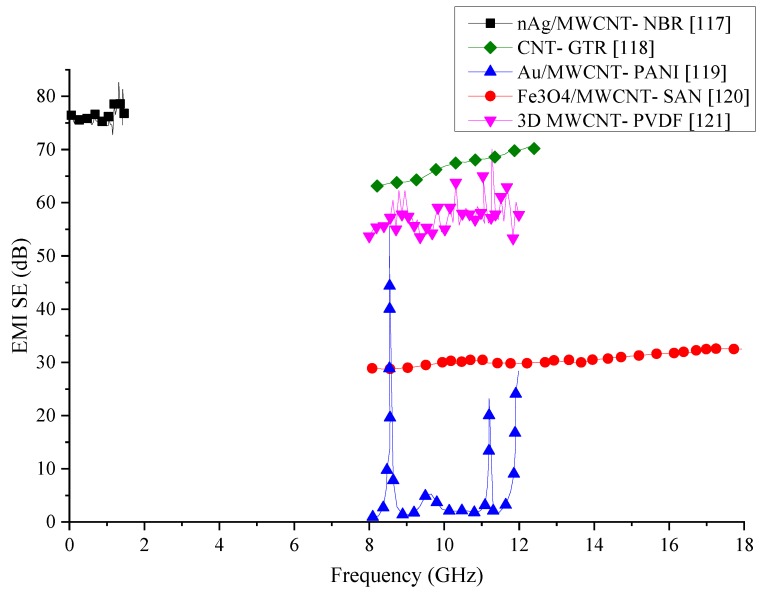
EMI SE comparison of reviewed polymer composites containing MWCNTs mixed with various fillers.

Many research aimed at fabricating novel materials for EMI shielding have shown that incorporating just one type of filler is insufficient to achieve the SE provided by existing metals. MWCNTs have shown they have excellent electrical conductivity and when combined with polymer matrices, have shown promising results for EMI SE. Combination of MWCNTs and other fillers that could enhance the SE have been investigated by recent research out of which some are reviewed in this section, and their details are summarised in Table 8. SE results of these composites have been compared graphically in Figure 23. From the comparison, nAg/MWCNT/NBR composite has shown the best shielding properties but within a small frequency range. The SE produced by this composite has fluctuated when the testing frequency is high. However, this is the only composite that has been able to produce a SE above 70 dB out of the publications reviewed in this section. One reason for the increased SE of this composite would be the presence of Ag, which is known to have extremely high electrical conductivity. The second best SE is produced by the composite containing CNT in GTR matrix. The SE of this composite is comparatively stable and above 60 dB. While CNT could enhance the SE of the composite from their high electrical conductivity, the presence of steel in GTR would help to extend the conducting network within the composite. Ferrite/MWCNT/SAN has also been able to generate a stable SE within the tested frequency range. However, the SE of this composite is in the range of 30 dB. Other composites containing CNTs mixed with other fillers have significant variations in their SE. Reasons for such significant variations in their SE have not been investigated by the authors. In order to create more stable SE, a variation of the fillers and matrix materials could have been investigated by the authors.

### 3.9. Metal Filler Added Polymer Composites

Addition of metals fibres to composites to enhance the SE has also been investigated by many researchers since metals are outstanding conductors, and they would contribute positively to the overall SE of the composite. Many metal fibres such as steel, copper, and silver have been added to composites with a not only polymer-based but also to other forms of matrices as well. In an attempt to make a lightweight and flexible composite with good SE, stainless steel (SS) fibres have been combined with polyester staple fibres to create a conductive yarn, which then has been woven to have different patterns. To assess the effect of metal fibres in the mixture, yarns with different metal loadings have been fabricated. EMI SE tests have been carried out in 8 to 18 GHz frequency range. Results have shown that when the stainless-steel fibres are arranged in the parallel direction, the reflection of the EMWs is higher than in the perpendicular direction. Moreover, with the increase in the density of the cloth, the SE also increases. A maximum SE of 31 dB has been achieving by these fabrics. However, since the thickness of the fabric is lower than most of the composites that have been tested for shielding properties, it takes a considerably large fraction of steel fibres to provide sufficient SE in this material, but this provides the basis for wearable EMI shielding materials [122]. 

**Figure 24:**
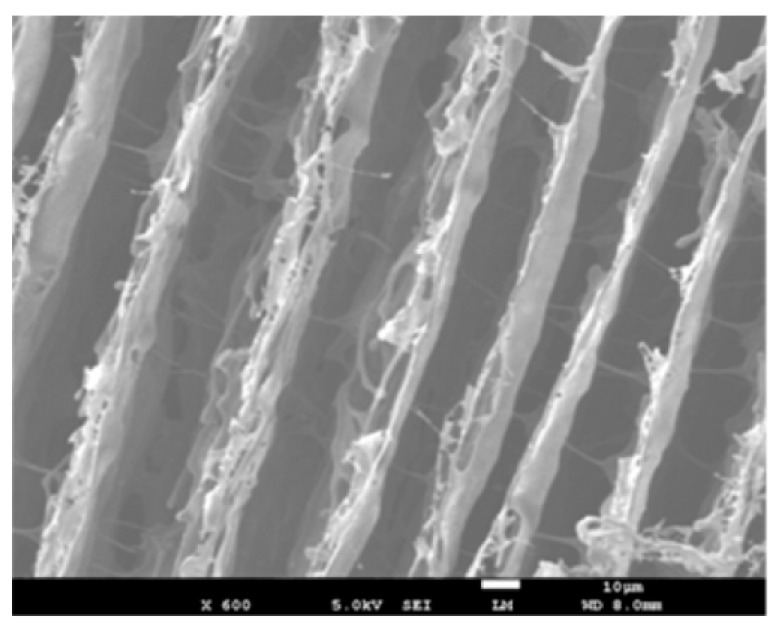
SEM image of Ag nanowire composite with 28.6 wt% Reproduced with permission from Zeng et al. [123], Copyright 2017, American Chemical Society.

In another research to fabricate flexible and lightweight composites for EMI shielding, silver (Ag) nanowires have been used as a reinforcement in a waterborne polyurethane (WPU) with a porous structure. Ag nanowires have been arranged in a parallel direction in this composites, and a variable amount of Ag nanowires have been added to assess the impact of them for the SE. The SEM image is shown in Figure 24, which indicates the direction of the nanowire arrangement within the composite. Micro-sized pores within the matrix have been obtained by the ice-templated freeze-drying method. Due to the presence of pores, the composite has shown very low density, and due to Ag nanowires, it has achieved high conductivity. The composite has been able to achieve a maximum SE of 64 dB within 8 to 12 GHz frequency range. The presence of porous structure has contributed to the absorption of the EMWs. Comparing to most of the thin, lightweight, and flexible EMI shielding composites, this particular one has shown outstanding SE in this frequency range. Additionally, it has also shown good mechanical properties and ease of fabrication compared to most of the other composites [123].

**Figure 25:**
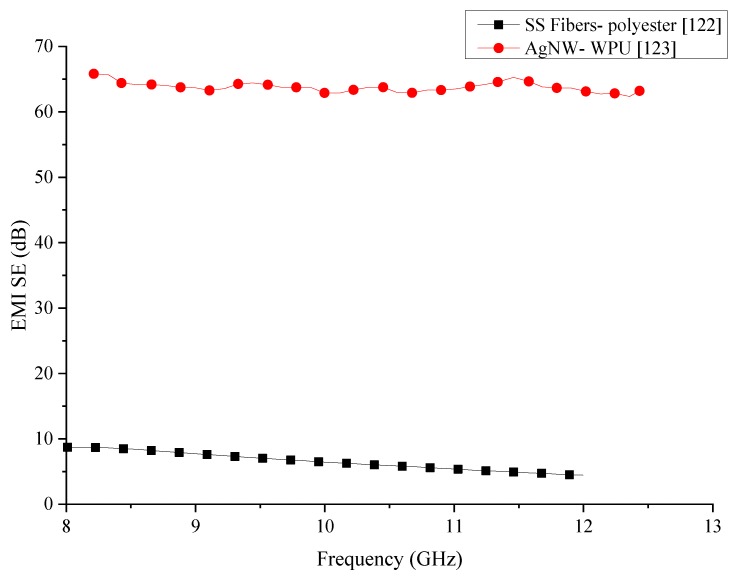
EMI SE comparison of reviewed polymer composites containing metallic fillers.

Information of the polymer composites with metal fillers which have been reviewed in this section are summarised in Table 9. In the comparison of the metal filler added polymer composites, which is shown in Figure 25, it can be seen that both composites have generated stable shielding properties within the same frequency range. However, the SS/polymer composite has considerably lower SE than the AgNW/WPU composite. Ag is one of the best conductor and the wires used in the fabrication of the composite are in nanoscale would have increased the overall conductivity of the composite. The increase of the electrical conductivity would result in higher EMI SE. However, the cost of the Ag nanowires is significantly higher than the SS fibres, which would result in the composite being extremely expensive. Addition of the metallic fibres to polymers is known to increase the mechanical properties of composites. There are no comparative data about the mechanical properties of the composites in the publications.

### 3.10. Particle Filler Added Polymer Composites

Various forms of particles are known to be good absorbers of EMWs and commonly used in the fabrication of composites. In addition to the increased SE of the composite, these particles can provide enhanced mechanical properties to the composite as well. In early research on the synthesis of composites for EMI shielding in the millimetre-wave region, titanium dioxides (TiO_2_) along with carbon particles have been mixed into an epoxy resin. Samples with various amounts of TiO_2_ and carbon particles were fabricated into a thin coating for testing. SE testing has been carried out in the 50 to 110 GHz frequency range. Samples with just 0.28 mm thickness were able to provide maximum absorption of 44.5 dB at a frequency of 96.8 GHz. Overall results from this experiment have shown that a composite with over 20 dB absorption can be fabricated by using the combination of these two particles for the studied frequency range [124].

**Figure 26:**
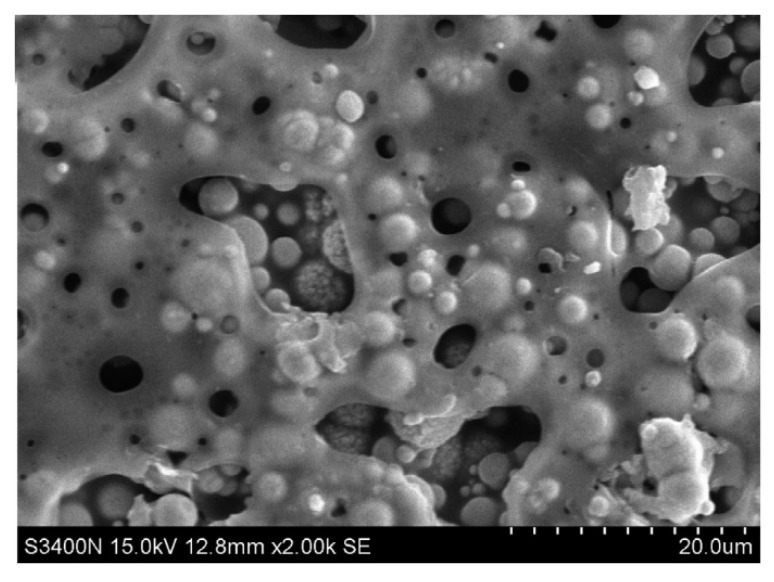
SEM image of surface of the electroconductive adhesive filled with silver-coated carbonyl iron powders. Reproduced with permission from Guo et al. [127], Copyright 2015, Elsevier Ltd.

Carbonyl iron powder (CIP) or extremely pure form of iron made with chemical deposition techniques have considerably good EMI shielding properties and are commonly used in composite manufacturing [125]. Early research into fabricating two EMW absorbing composites consisting of CIP involved the addition of 10% CIP powder to epoxy and silicon rubber matrices. The fabricated composites have been tested within 26 to 40 GHz frequency range. Within this frequency range, these composites have been able to produce a SE of 8 to 12 dB and 8 to 11 dB respectively. Moreover, the SE of both of the composites has reduced with the increase of the frequency [126]. To improve the conductivity of the CIP and hence improve the SE of the composite, a silver coating has been applied on the particles. The synthesised composite with an epoxy matrix has been tested in the frequency range of 100 kHz to 1.5 GHz. Ag coating of the CIP has been carried out by a chemical route, and they have been characterised by various methods to confirm the success of the process. Image obtained during the SEM analysis on the synthesised composite is shown in Figure 26, indicates the thorough distribution of the coated powder within the matrix. The SE test has shown that this composite can have a SE above 38 dB. The Ag coating on the powder has increased the overall SE of the composite by increasing the multiple reflections, thus attenuating the EMWs [127].

One of the primary purposes of using a polymeric matrix to fabricate a composite for EMI shielding is to reduce the density of the composite. While the addition of fillers can increase the SE, it can also affect the density of the composite. The addition of metallic or metal-based fillers increases the density of the composite, making it heavier. Therefore, the addition of more lightweight fillers has been investigated by some researchers. Many of these lightweight fillers include conductive polymers such as PPy. Since the conductivity of PPy is not as high as that of metals, Ag nanoparticles have been used to enhance the conductivity of this filler. A Ag nanoparticles-coated hollow PPy microsphere composite has been investigated for its SE with different Ag loading in the frequency range of 0.5 to 8 GHz. The best shielding performance has been reported by 10 wt% Ag loaded composite which has been 23 to 59 dB. Mechanism of shielding of the EMWs has been a reflection and multiple reflections [128]. Since Ag is an excellent conductor, it is commonly used as a coating for many other particles to enhance their conducting properties [129,130]. Different fractions of Cu core shells coated with Ag (Cu@Ag) have been used in fabricating a lightweight and flexible EMI shielding composite with a leather matrix that can be worn. The composite with 5.17 vol% of filler has been able to produce a SE close to 100 dB within 10 to 3000 MHz frequency range. The thickness of the tested specimens has been only 0.6 mm, which indicates an excellent SE from the composite, and the primary shielding mechanism has been reflection [40].

Many-particle reinforced composites have been investigated for the EMI shielding properties in numerous research, including perovskites particles such as La_0.8_Ag_0.2_MnO_3_ particles. The composite containing 20 wt% of these perovskites particles and a paraffin matrix has been tested for the SE within the 1 to 18 GHz range. The thickness of the specimen was 2 mm. The composite has been able to produce a SE of 10 to 36 dB within this frequency range. The SE has increased with the frequency, and the primary shielding mechanism has been the absorption of the EMWs [131]. Another composite made with Li_0.5_Fe_2.5-*x*_Gd*_x_*O_4_ (*x* = 0.0, 0.05, 0.10, 0.15, and 0.2) ferrite nanoparticle fillers and PANI matrix has been tested for its SE within 8 to 12 GHz frequency range. The composite has shown an overall SE of about 41 to 42 dB within this frequency range, with absorption being the main shielding mechanism. As expected, the maximum SE has been obtained in the composite with the maximum amount of fillers [132]. 

**Figure 27:**
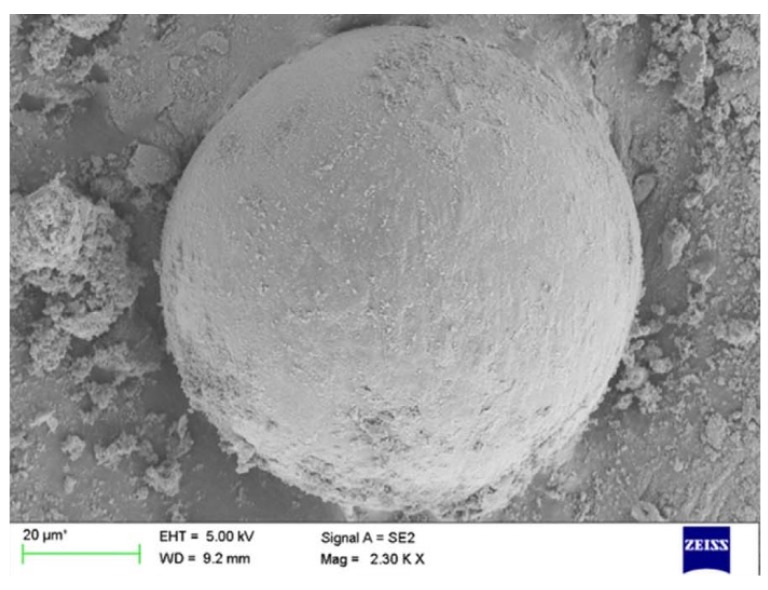
SEM image of NiO coated cenospheres used in PNiOC. Reproduced with permission from Bora et al. [133], Copyright 2017, Elsevier Ltd.

A thin-film composite (PNiOC) consisting of NiO coated fly ash cenospheres, and PANI as the matrix have been fabricated and tested for SE within 5.8 to 8.2 GHz, 8.2 to 12.4 GHz, and 12.4 to 18 GHz frequency ranges. SEM image of the particles shown in Figure 27, shows that the fly ash particles to be spherical. The average thickness of the specimen has been 81 ± 3 μm. For each frequency ranges the composite has been able to produce an average SE of about 24 dB, 27–24 dB, and 21 dB respectively. While the main shielding mechanism has been identified as the absorption, the synergetic effect between the fillers and the matrix have also contributed to the high SE of the composite. In addition to the good SE shown by the composite, the cost of the composite is also reported to be low compared to most polymer-based composites. These thin films make it ideal to be used in miniature electronic devices such as mobile phones [133]. 

Ferrite is one of the most commonly used particles in the preparation of composites for EMI shielding applications. A composite consisting of ferrite nanoparticles mixed with polyvinylpyrrolidone (PVP) matrix has been tested for the SE within 8.2 to 12.4 GHz frequency range. The ferrite content of the composite has been only 4 wt%, and the thickness of the tested specimen has been 1 mm. The SE produced by this composite has been 22 dB. Compared with previous literature work of composites using ferrite as fillers, the authors have found that the SE produced by their composite to be a considerably high value with such a low loading of fillers [134].

**Figure 28:**
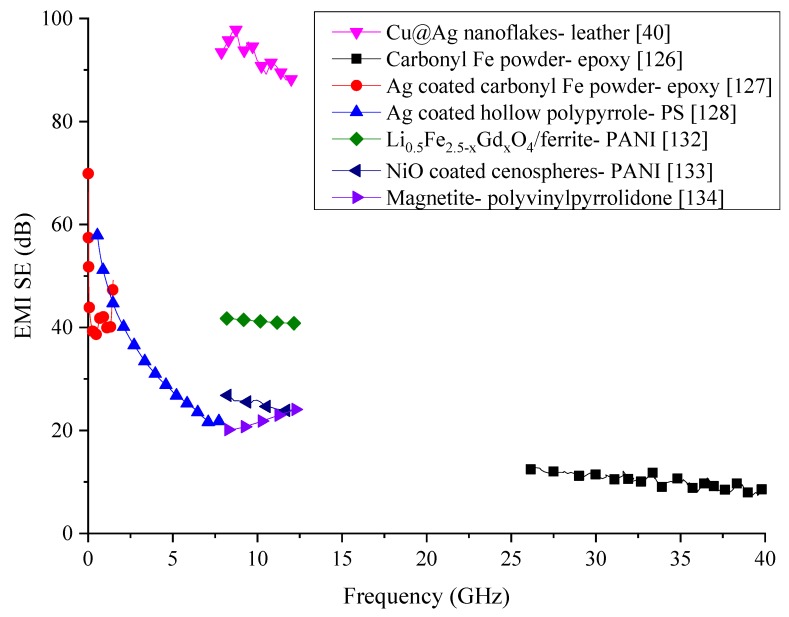
EMI SE comparison of reviewed polymer composites containing particulate fillers.

Many of the particle-based composites have shown that their EMI shielding properties arise as a result of either the EMWs undergoing multiple reflections within hollow particles or reflection due to the surface conductivity. There have been many research into particle filler based EMI shielding composites since these particles utilised can act as EMW absorbers while extending the electrical conducting network within the composite by acting as nodes. Details of the composites reviewed under this section are summarised in Table 10. Many of the composites that are being researched for EMI shielding utilise nanoparticles as they have a large surface area compared to their micro counterparts. Previous research conducted on composites with nanoparticles have shown to have superior properties to composite with microparticles. Many of the composites reviewed within this section have particles coated with a secondary material to improve the reflection and multiple reflections of EMWs. Ag seems to be the most commonly used coating material on these particles as the high conductivity of Ag would enhance the conducting network within the composite leading to high SE. Comparison of the composites analysed in this section is provided in Figure 28. The best SE from the reviewed composites within this section has been generated by the Cu@Ag nanoflakes embedded leather composite. The primary objective of this research has been to fabricate a flexible and wearable composite that would protect the wearer from EMI. Comparative to many of the composites that are being designed for EMI shielding, this composite has shown remarkably high SE and has been able to achieve the objectives of the research. However, authors report that the composite might require further improvements in terms of its fabrication process and durability. All the other composites reviewed in this section have considerably lower SE. Composites containing carbonyl iron powder has shown the lowest SE in this section with the composite containing magnetite particles faring behind. Ag coated carbonyl iron powder based composite has shown much better EMI performance but within a lower frequency range. Additionally, the composite has shown a large variation in its SE within the tested frequency range. Ag coating has shown to improve the overall SE of the composite with the Ag coated polypyrrole/PS composite also showing promising results. The SE of this composite has shown a rapid decrease with the increase of the composite, which might be avoided if a secondary filler is added to absorb the EMWs with higher frequency. The LiFeGdO/Ferrite/PANI composite has shown somewhat steady SE within the tested frequency range hold promising results that it could be developed to enhance its SE further to meet the industry standards.

### 3.11. MXene-Based Polymer Composites

MXene is a two-dimensional ceramic material, which consists of several layers of nanoscale titanium-aluminium carbides in different proportions. However, different forms of arrangements of layers could be achieved in MXene by varying the type of transition metal that is being used [135]. MXene is commonly used in composites which requires high electrical conductivity. Hence, there is a growing interest in using MXene for a composite that could be used for EMI shielding applications. Using a polymeric matrix with the combination of MXene have proven to increase the electrical conductivity of the composite drastically.

In one of the most recent works with MXene, Raagulan et al. have fabricated a polymer called PAT polymer consisting of MXene with poly(*p*-aminophenol) (PpAP) and polyaniline–PpAP (PANI–PpAP) conductive polymers. In addition to these polymers, authors have also used other conductive polymers to compare the effect of the matrix on the overall SE of the composites. The fabrication processes used for each composite fabrication have been details in their original work, and they have utilised various characterisation techniques to assess the properties and morphology of the fabricated composites. EMI shielding properties have been measured in a frequency range of 8.2 to 12.4 GHz. The maximum SE of 45.18 dB has been produced by a composite consisting of MXene–PAT–PANI–PpAP composite [136].

Wan et al. have employed MXene with poly(3,4-ethylenedioxythiophene)/poly(styrene- sulfonate) (PEDOT/PSS) for the fabrication of a composite with high EMI shielding and mechanical properties. The specimens have been subjected to EMI shielding tests in 8.2–12.5 GHz and 11.9–18 GHz frequency ranges. A composite, with a thickness of 6.6 μm, has been able to generate a maximum SE of 40.5 dB within these frequency ranges. In addition to this impressive SE, the fabricated specimens have also shown a tensile strength of 38.5 ± 2.9 MPa, which is a significantly higher value when compared to other polymer composites with MXene [137].

In an attempt to make a flexible composite with high EMI shielding properties, Hu et al. have utilised MXene with cellulose employing a low-cost dip-coating manufacturing method. In addition to EMI shielding properties, authors have aimed to achieve high thermal conductivity in this polymer as well. Details description of the low-cost manufacturing technique they used can be found in their original work. Fabricated specimens, with a thickness of 0.2 mm, have been subjected to EMI shielding tests within a frequency range of 8.2–18 GHz. Results have shown that the composites can generate an EMI SE of 43 dB in the tested frequency range in addition to high electrical and thermal conductivity, which is higher than many of the polymer composites using MXene [138].

The high electrical conductivity of MXene layers has made it a very attractive additive to be used in the fabrication of polymer-based composites for EMI shielding. Currently, there are a large number of publications of polymer composites with MXene as the main additive fabricated for EMI shielding. The nanoscale thickness of the MXene makes these composite an excellent choice for EMI shielding applications where flexibility and low thickness are required [139,140,141]. Summary of these composites containing MXene in different forms is provided in Table 11.

## 4. Summary

**Figure 29:**
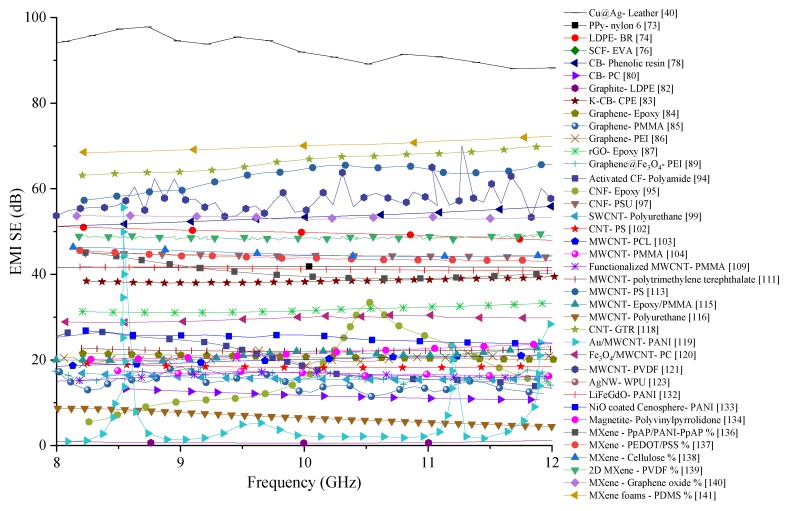
EMI SE comparison of reviewed polymer composites within 8 to 12 GHz frequency range.

Many of the composites reviewed in this paper have been characterised within the 8 to 12 GHz frequency range. While there are some composites which have been tested outside of this range, the comparison shown in Figure 29 has been carried out for the most commonly used frequency range used for the characterisation of most of the composites. From the first glance of the comparison, it can be seen that many composites cannot generate SE above 70 dB except for the Cu core shells coated with Ag (Cu@Ag) embedded leather which has remarkable high SE compared to other composites. The second best SE within this frequency range has been produced by the composite with CNT/GTR composite, followed by MWCNT/PS and MWCNT/PVDF composites. The high electrical conductivity of MWCNTs may have contributed to the comparative high SE. However, composites with PANI matrices have not been able to generate high SE even though PANI is one of the polymers with better electrical conductivity. Regardless of the type of filler used, the majority of the polymer-based composites generate a SE of only about 20 dB. Analysing the type of filler that is added in the polymer composites with high SE, it can be concluded that very high electrical conductivity is necessary for the composite to generate high shielding properties. The addition of high conducting fillers is not the only factor that has been contributed to the high SE. Other factors such as additional fillers, filler distribution, and extension of the conducting network also are significant factors that have shown to increase the EMI SE dramatically rather than the addition of high conductive fillers alone.

**Figure 30:**
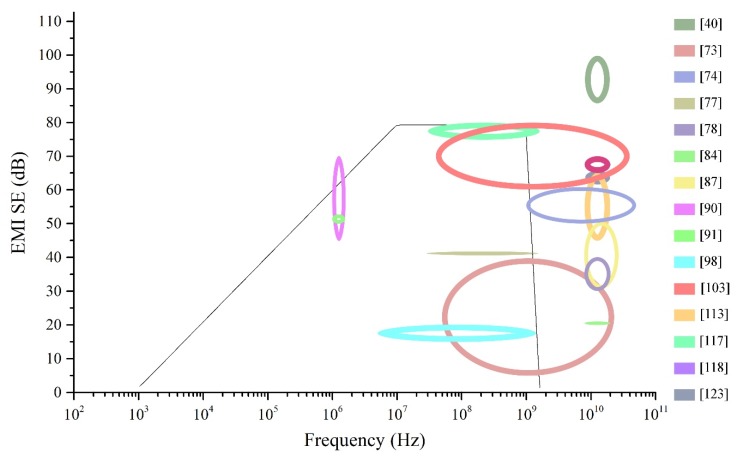
EMI SE distribution of polymeric composites having high SE compared to SE requirement of MIL-STD-188-125-1.

The distribution of EMI SE in polymeric composites reviewed in this paper has been compared with the SE requirement described in MIL-STD-188-125-1, which is shown in Figure 30. The graphical comparison shows that most of the polymeric composites have been developed for high-frequency applications that are not defined in the MIL-STD-188-125-1. Most of the composites that have been tested in the high frequency have surpassed the minimum shielding requirement defined by the standard. However, the highest SE is again shown by the Cu@Ag/leather composite, which is significantly higher than any other composite. The comparison also shows that many of the composites have been tested in a very narrow frequency range, and some of them have produced very low SE while some have been able to produce moderately high SE. In order to carry out a more detailed analysis of each of the composites regarding the SE they produce, they need to be tested in a wider frequency range. Since MIL-STD-188-125-1 has been defined for the SE needed in buildings, many of the polymeric composites that are being developed need to focus on lower frequency EMWs if they are to be used for similar applications. The distribution of EMI shielding provided by these composites shows that they could be developed to meet the requirement of the standard with further developments.

## 5. New Developments

Most of the novel studies into EMI shielding materials have been focused on synthesising lightweight materials, which makes polymer matrix composites an ideal choice. Low cost and ease of fabrication also are advantages when selecting polymer-based composites. In some of the latest published literature there have been studies looking into orienting continuous carbon fibres in a polyamide nylon-6 matrix composite, incorporating metallic nanowires with controllable morphologies within a polyvinylpyrrolidone matrix, MWCNT mixed with various other particles and fibrous fillers, layered composites consisting of conductive materials, etc. [142,143,144,145,146,147]. One of the critical factors in most of these up and coming studies is the addition of multiple fillers. The main reason for the addition of multiple fillers is that the addition of just one type of filler is often insufficient to achieve the SE required by industry. Another essential manufacturing process which is yet to be utilised for materials designed for EMI shielding is 3D printing technology. Although there has been much development in 3D printing technologies and EMI shielding materials, the two fields are yet to be combined. However, in a recent publication, 3D printing has been used indirectly to synthesize an EMI shielding material, in which the hollow microporous structure has been fabricated by using this rapid prototyping technology [148]. Developments in metallic materials are also being published recently but will not as high frequency as polymer-based materials. In most of the metal-based material studies, one key ingredient has been the MXenes, mainly due to their high electrical conductivity [149]. Other metal-based metallic material research has focused on either creating hollow metallic materials or incorporating metal oxides [150]. Compared to polymeric and metallic-based shielding materials, cementitious materials represent a very small amount of the recent publications. Many of the published works on cementitious materials make use of metallic fibres in their mix due to the ease of breakage of of the fragile fibres which are used in polymeric materials. 

## 6. Conclusions

The development in the electronics industry have pushed EMI shielding requirements to new heights in the past decade. To meet this demand, there have been a large number of new studies focusing on fabricating new materials that can provide the same amount of shielding while eliminating all the drawbacks of classically used metallic shields. Many of these new studies are branched depending on the type of matrix material that is being used to fabricate them. Since the extremely high SE of metals is generated by their high electrical conductivity properties, many of the studies on alternative materials are also focused on increasing the electrical conductivity of the composites that are being studied for their SE. Polymer matrix composites have attractive alternative materials that are being studied for EMI SE. The many advantages offered by the polymers compared with other categories of materials have been the main reason for this increased interest. However, despite their numerous advantages, polymers fail to provide high SE due to their inherent low electrical conductivity properties. Hence, many of the researchers have attempted to increase the electrical conductivity of polymer composites with the addition of different types of conductive fillers. However, results from various polymer composite studies have shown that the SE of polymer matrix composites does not depend solely on the conductivity of the matrix and a considerable amount of EMWs can transmit through the material.

This paper has reviewed various polymer matrix composites that have been fabricated for EMI shielding. While increasing the electrical conductivity have been able to increase the SE provided by polymeric composites that alone is not enough to generate the SE needed in practical applications. As a result, experiments on fabricating polymeric composites for EMI shielding have expanded to including nano and multiple fillers. The inclusion of multiple fillers has shown to increase the SE of composites by absorbing and subjecting EMWs to multiple reflections. This is evident from the SE results of conductive polymer composites in this review. Even with the matrix being conductive, the overall SE produced by these composites has been very low. On the other hand, the addition of MWCNTs and CB have shown better results indicating that a good conducting network is mandatory for generating high SE.

A majority of the composites that have been reviewed in this work have been able to generate a SE of about 20 dB, which is not sufficient to meet the standards required in industry. Few of the composites have been able to generate high SE. Composites with extremely high SE have been fabricated with high conductive Cu core shells coated with Ag (Cu@Ag) particles. With the high flexibility provided by this composite, there is a promise that it could be developed to cater to industry requirement. Ag/MWCNT composite has also been able to generate very high SE in the tested frequency range, which also leads to the conclusion that very high conductive network is needed to generate sufficient SE in polymer matrix composites. 

While many of the studies have focused on creating high SE in polymeric composites, there has been a minimal focus on mechanical and fire-retardant properties. Since components used for EMI shielding also will need to bear mechanical loads during operation, it is essential that these composites should be tested for their mechanical properties too. However, only a handful of the composites in this review have had their mechanical properties tested. Additionally, since polymers are known to be flammable, the thermal and fire-retardant properties need to be investigated, which have not been tested by any of the research reviewed here.

The EMI SE requirements defined by the US Department of Defense and other industry standards have a specific frequency range for the shielding requirements. Many of the composites reviewed in this work have focused on 8 to 12 GHz frequency range. Few other composites have been tested beyond this frequency range, which is summarised in each section in this review. From the results analysed in this review, it is clear that so far, there is no polymer composite that can replace the metal shields being used currently. This leads to further research into polymer matrix composites with mixed fillers. Since many of the composites that have been able to generate high SE have 3D conductive network, it can be concluded that multiple fillers with high electrical conductivity are necessary to create a polymeric composite with high SE.

## Figures and Tables

**Table 1:** Summary of electrically conductive EMI shielding polymer composites.

No.	Material-Filler	Material-Matrix	Frequency	Specimens Thickness	Effect of Shielding	Reference
1	Conducting polymer-coated fabrics	Fabric	101 GHz	-	35.61 dB	[72]
2	Polypyrrole (PPy)	Nylon 6	50 MHz–13 GHz	-	5–40 dB	[73]
3	Low-density polyethylene (PE)	Butyl rubber (IIR)	1–15 GHz	-	50–61 dB	[74]

**Table 2:** Summary of carbon black added EMI shielding polymer composites.

No.	Material-Filler	Material-Matrix	Frequency	Specimens Thickness	Effect of Shielding	Reference
1	Conductive carbon black (Vulcan XC-72)/short carbon fibre (SCF)	Natural rubber/ethylene vinyl acetate	100–2000 MHz/8–12 GHz	1.8 mm/3.5 mm	16–18, 25/26–30, 30–35 dB	[75]
2	Carbon black/short carbon fiber (SCF)/MWCNTs	Ethylene vinyl acetate copolymer	7.8–12.4 GHz	-	11–21 dB/31–41 d B/4–6 dB	[76]
3	Carbon black/synthetic graphite particles/milled pitch-based carbon fiber	Nylon 6,6/polycarbonate	30 MHz–1.5 GHz	-	41–42 dB/17–19 dB	[77]
4	Carbon black (CB) nanoparticles	Phenolic resin	8–12 GHz	-	30–40 dB	[78]
5	General purpose furnace (GPF) carbon black	Natural rubber NR/Butyl rubber (70/30)	0.5–5 GHz	2 mm	7–30 dB	[79]
6	Carbon black (CB)/carbon nanotubes (CNT)/graphene nanoplatelets (GNP)	Polycarbonate (PC)	8.5–12 GHz	22.86 mm × 10.16 mm	12 dB (max)/13 dB (max)/11 dB (max)	[80]
7	Natural microcrystalline graphite	Low-density polyethylene	2–18 GHz	2.0–2.1 mm	less than −10 dB comes to 3.02 GHz, and the reflectivity peak is of −20.46 dB	[82]
8	Ketjen carbon black (K-CB)	Chlorinated polyethylene (CPE)	8.2–12.4 GHz	1 mm	38.4 dB (at 30 wt% loading)	[83]

**Table 3:** Summary of graphene mixed EMI shielding polymer composites.

No.	Material-Filler	Material-Matrix	Frequency	Specimens Thickness	Effect of Shielding	Reference
1	Graphene	Epoxy	8.2–12.4 GHz	-	20–21 dB	[84]
2	Graphene	Polymethylmethacrylate (PMMA)	8–12 GHz	4 mm	13–19 dB	[85]

**Table 4:** Summary of EMI shielding polymer foams.

No.	Material-Filler	Material-Matrix	Frequency	Specimens Thickness	Effect of Shielding	Reference
1	Graphene	PEI	8–12 GHz	2.3 mm	7.27–19.66 dB	[86]
2	Graphene@Fe_3_O_4_ (G@Fe_3_O_4_) particles	PEI	8–12 GHz	2.5 mm	∼14.3–18.2 dB	[87]
3	Reduced graphene oxide coated carbon fiber (rGO-CF, GCF) and Fe_3_O_4_ nanoparticles deposited rGO nanohybrids (magnetic graphene, MG)	Epoxy	8.2–12.4 GHz, 12.4–18.0 GHz, 18.0–26.5 GHz	7 mm	31.3–51.1 dB	[89]

**Table 5:** Summary of carbon fibre mixed EMI shielding polymer composites.

No.	Material-Filler	Material-Matrix	Frequency	Specimens Thickness	Effect of Shielding	Reference
1	Carbon fiber	Nylon-6,6	1–1500 MHz	1.2 mm	45–70 dB	[90]
2	Carbon-fiber	Liquid-crystal polymer (LCP)	1–1500 MHz	10 mm	50–53 dB	[91]
3	Activated carbon fibers	epoxy	1.0–1.5 GHz	3 mm	39 dB	[92]
4	Activated carbon fibre	Polyamide resin	2–18 GHz	4 mm	−10 to −26.8 dB	[93]
5	Carbon nano fibres	Epoxy	8–20 GHz	1, 2, and 3 mm	−18.3 dB (1 mm thick)/−10.8 dB (3 mm thick)	[94]
6	Carbon Nanofiber	Epoxy	8.2–12.4 GHz	2.1 mm	−10 to −34 dB	[95]
7	Carbon nanofiber (CNF)	Hollow carbon microspheres (HCMs) and resole resin	30 MHz to 1.2 GHz	-	25 dB	[96]
8	Carbon nanofibers	Polysulfone (PSU)	8.2–12.4 GHz	1 mm	19–45 dB	[97]

**Table 6:** Summary of polymer-based shielding materials.

No.	Material-Filler	Material-Matrix	Frequency	Specimens Thickness	Effect of Shielding	Reference
1	SWCNTs	Epoxy	500 MHz–1.5 GHz	1.5 mm	15–20 dB	[98]
2	SWCNTs	Polyurethane	8.2–12.4 GHz	2 mm	~17dB	[99]
3	SWCNT bundles/MWCNT/Arc discharge CNT/Carbon foam	Epoxy/Epoxy/Epoxy/NA	30 GHz	-	4.8 dB/3.7 dB/2.0 dB/9.0–23.4 dB	[100]

**Table 7:** Summary of polymer-based shielding materials.

No.	Material-Filler	Material-Matrix	Frequency	Specimens Thickness	Effect of Shielding	Reference
1	MWCNTs with graphite crystal structure	Poly(vinylidene fluoride) (PVDF)/poly(vinyl pyrrolidone) (PVP)	10–1500 MHz	400–600 μm	17–21 dB	[101]
2	Carbon nanotube	Polystyrene foam	8.2–12.4 GHz	-	18.2–19.3 dB	[102]
3	MWCNTs	polycaprolactone (PCL)	40 MHz–40 GHz	2 cm	60–80 dB	[103]
4	MWCNTs	PMMA/PS	8.0–12 GHz	0.2–0.3 mm	18 dB	[104]
5	MWCNTs	PANI	12.4–18.0 GHz	2 mm	−27.5 to −39.2 dB	[105]
6	MWCNTs	Epoxy	800 MHz–4 GHz	2 mm	22–32 dB	[106]
7	Functionalized (maleic anhydride modified) MWCNTs	Poly (methyl methacrylate)	2–18 GHz	1 mm	13–18 dB	[109]
8	MWCNT	Poly(trimethylene terephthalate) [PTT]	12.4–18 GHz	2 mm	36–42 dB	[110]
9	MWCNTs	Poly (trimethylene terephthalate)	8.2–12.4 GHz	2 mm	22 dB	[111]
10	MWCNTs	Polycarbonate	8.2–12.4 GHz	1.85 mm	25 dB	[112]
11	MWCNTs	PS	8.2–12.4 GHz	2 mm	45 dB/65 dB	[113]
12	MWCNTs	Polyhedral oligomeric silsesquioxane (POSS)	36–50 GHz	0.4 mm	15–16 dB	[114]
13	MWCNTs	Epoxy composites and PMMA coatings	100 MHz–14 GHz	100 μm	20 dB	[115]
14	MWCNTs	Polyurethane the cured paint was applied on Laminated composite with 10 alternating layers of continuous GF and PPS	8–12 GHz	0.15 mm	Reflection −9 dB to −5.5 dB (increasing with frequency)	[116]

**Table 8:** Summary of EMI shielding polymer composite with MWCNTs with mixed fillers.

No.	Material-Filler	Material-Matrix	Frequency	Specimens Thickness	Effect of Shielding	Reference
1	Microscale silver flakes (Ag flakes), MWCNTs decorated with nanoscale silver particles (nAg-MWNTs)	Nitrile butadiene rubber (NBR)	30 MHz–1.5 GHz	Cylindrical rod with a radius of 4 mm	~45 dB	[117]
2	CNTs 7000 series	Ground tire rubber (GTR)	8.2–12.4 GHz	2.6 mm	66.9 dB	[118]
3	Au-MWCNTs	Polyaniline	8–12 GHz	2 mm	Reflection loss −16 (max) and −56.5 (min) dB	[119]
4	Dopamine anchored iron oxide (Fe_3_O_4_) nanoparticles with chemically grafted MWCNTs	PC/SAN [poly (styrene-co-acrylonitrile)] blend	8.2–12 GHz and 12–18 GHz	-	−32.5 dB at 18 GHz for 3 wt% loading	[120]
5	MWCNT 3D network	PVDF	8.2–12.4 GHz	2 mm	56.72 dB	[121]

**Table 9:** Summary of metal filler incorporated EMI shielding polymer composites.

No.	Material-Filler	Material-Matrix	Frequency	Specimens Thickness	Effect of Shielding	Reference
1	Stainless steel fiber	Polyester	8–18 GHz	NA Specimens varied based on the weaving pattern	31 dB	[122]
2	Silver nanowire (AgNW)	Waterborne polyurethane (WPU)	8.2–12.4 GHz	2.3 mm	64 dB	[123]

**Table 10:** Summary of polymer-based shielding materials.

No.	Material-Filler	Material-Matrix	Frequency	Specimens Thickness	Effect of Shielding	Reference
1	Cu@Ag nanoflakes	Leather	8–12 GHz	-	100 dB	[40]
2	Titanium dioxide and carbon particles	Epoxy resin containing carbon particles	50–110 GHz	2 mm	More than 20 dB	[124]
3	Carbonyl iron powder	Epoxy/silicon rubber	26–40 GHz	5 mm and 10 mm	8–12 dB/8–11 dB	[126]
4	Silver-coated carbonyl iron powder	Epoxy	100 kHz–1.5 GHz	0.35 mm	Above 38 dB	[127]
5	Hollow polypyrrole coated with Ag nanoparticles	Polystyrene (PS)	100 KHz–20 GHz	-	34.5–6 dB	[128]
6	La_0.8_Ag_0.2_MnO_3_	Paraffin	1–18 GHz	8.5 mm	36 dB	[131]
7	Li_0.5_Fe_2.5-x_Gd_x_O_4_ (0.0 ≤ x ≤ 0.2) ferrite nanoparticles	PANI	8–12 GHz	2 mm	SE_A_ = 34−36 dB, SE_R_ = 4.0−6.3 dB, SE_T_ = 42 dB	[132]
8	NiO coated cenosphere	Polyaniline (PANI)	5.8–8.2 GHz/8.2–12.4 GHz/12.4–18 GHz	84 ± 2 μm/80 ± 3 μm/81 ± 3 μm	~24 dB/~27–24 dB/~21 dB	[133]
9	Magnetite nanoparticles	Polyvinylpyrrolidone	8.2–12.4 GHz	-	22 dB	[134]

**Table 11:** Summary of MXene-based shielding materials.

No.	Material-Filler	Material-Matrix	Frequency	Specimens Thickness	Effect of Shielding	Reference
1	MXene and reduced MXene	Poly(p-aminophenol) (PpAP) and Polyaniline–PpAP (PANI–PpAP) conductive polymers	8.2 to 12.4 GHz	0.4–1.6 mm	45.18 dB	[136]
2	MXene sheets	Poly(3,4-Ethylenedioxythiophene)/Poly(styrenesulfonate) (PEDOT/PSS)	8.2–12.5 GHz/11.9–18 GHz	~6−~9 μm	40.5 dB	[137]
3	MXene	Cellulose	8.2–18 GHz	0.2 mm	43 dB	[138]
4	2D MXene	Polyvinylidene fluoride (PVDF)	8.2–12.5 GHz	2 mm	48.47 ± 3.5 dB	[139]
5	MXene films	Graphene oxide	8.2–12.4 GHz	7 μm	50.2 dB	[140]
6	MXene foams	Polydimethylsiloxane (PDMS)	8.2−12.4 GHz	2 mm	70.5 dB	[141]
